# Probing the Effects of Electrode Composition and Morphology on the Effectiveness of Silicon Oxide Overlayers to Enhance Selective Oxygen Evolution in the Presence of Chloride Ions

**DOI:** 10.1021/acs.jpcc.2c07116

**Published:** 2022-11-22

**Authors:** Johannes G. Vos, Amar A. Bhardwaj, Adriaan W. Jeremiasse, Daniel V. Esposito, Marc T. M. Koper

**Affiliations:** † Leiden Institute of Chemistry, Leiden University, PO Box 9502, 2300 RA Leiden, The Netherlands; ‡ Magneto Special Anodes (an Evoqua brand), Calandstraat 109, 3125 BA Schiedam, The Netherlands; § Department of Chemical Engineering, Columbia Electrochemical Energy Center, Lenfest Center for Sustainable Energy, Columbia University in the City of New York, 500 W. 120th Street, New York, New York 10027, United States

## Abstract

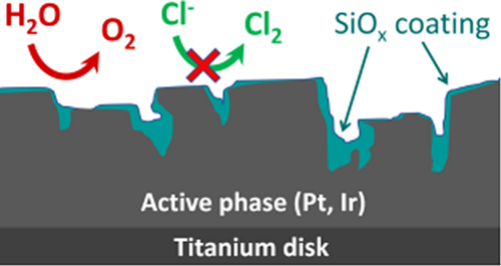

Seawater electrolysis offers significant logistical advantages over freshwater electrolysis but suffers from a fundamental selectivity problem at the anode. To prevent the evolution of toxic chlorine alongside the evolution of oxygen, a promising approach is the use of electrochemically inert overlayers. Such thin films can exert a perm-selective effect, allowing the transport of water and oxygen between the bulk electrolyte and the electrocatalytic buried interface while suppressing the transport of chloride ions. In this work, we investigate thin (5–20 nm) overlayer films composed of amorphous silicon oxide (SiO*_x_*) and their application to suppressing the chlorine evolution reaction (CER) in favor of the oxygen evolution reaction (OER) during acidic saltwater electrolysis on three different types of electrodes. While SiO*_x_* overlayers are seen to be an effective barrier against the CER on well-defined, smooth Pt thin films, decreasing the CER activity roughly 20-fold, this ability has not been previously explored on Ir-based catalysts with a higher surface area relevant to industrial applications. On amorphous iridium oxide electrodes, the selectivity toward the CER versus the OER was marginally reduced from ∼98 to ∼94%, which was attributed to the higher abundance of defects in overlayers deposited on the rougher electrode. On the other hand, Ir-based anodes consisting of thick mixed metal oxide films supported on Ti showed a significant decrease in CER selectivity, from ∼100 to ∼50%, although this came at the cost of reduced activity toward the OER. These results show that the morphology and composition of the underlying electrode play important roles in the effectiveness of the selective overlayers and provide guidance for further development of high-surface-area OER-selective anodes.

## 1 Introduction

Hydrogen production from water electrolysis is a promising method to offset the inherent intermittency of renewable energy sources, such as wind or solar.^1−3^ It is based on the conversion of electrical energy into chemical energy by generating H_2_ at the cathode; to complete the device, the ideal anodic reaction is the production of O_2_ via the oxygen evolution reaction (OER).^4−6^Equation 1 describes the OER in acidic/neutral media
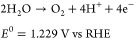
1

The O_2_ is considered an environmentally harmless byproduct that can be conveniently discarded into the atmosphere. When water electrolysis is implemented at a large scale, saline water forms a more attractive feedstock than freshwater, since the potential of solar or wind energy is generally highest in arid regions and/or at sea or in coastal areas.^7−14^ Unfortunately, direct use of seawater in electrolyzers is hindered by anodic side reactions related to chloride, which is present in seawater in high concentrations (∼0.6 M). Chloride may react to form Cl_2_ through the chlorine evolution reaction (CER), according to
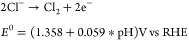
2

Depending on the solution pH, Cl_2_ may react further to form hypochlorous acid and hypochlorite; all of these are labile oxidizing species that form an environmental hazard.

To prevent the electrochemical generation of Cl_2_ during saline water electrolysis, the development of OER-selective anodes is of central importance. Avoiding the CER is not an easy task as it has an inherent kinetic advantage over the OER; the two-versus four-electron nature of the CER and the OER, respectively, implies that the latter has much slower kinetics and is a more difficult reaction to catalyze.^15−17^ This problem is aggravated by the existence of a scaling relationship between the OER and CER, which has been reported by both theory and experiment.^18−22^ The positive correlation between OER and CER activity has been attributed to similarities in how reaction intermediates related to oxygen and chlorine bind to catalyst surfaces^23−26^ and implies that any OER-active catalyst has the latent ability to catalyze the CER. Reaching high OER selectivity on the basis of kinetic principles alone may thus be very difficult, if not impossible.

One may prevent chloride oxidation by increasing the solution pH such that it becomes thermodynamically disfavored versus the OER.^7^ However, this concept places strict limits on the maximum anode potential that can be employed during electrolysis, lest chloride is still oxidized. A complicating factor is that the OER lowers the pH at the anode (see eq 1) so that under high OER current densities, the local environment near the anode surface may acidify enough to favor the CER, even in strongly alkaline solutions. The danger of evolving chlorine will then always loom over the process. It is therefore desirable to eliminate the possibility of chloride oxidation altogether.

An alternative approach to suppressing the CER is to steer the selectivity between competing reactions by applying a semipermeable, electrochemically inert coating on the catalytic surface.^27,28^ Such coatings can selectively impact the transport of electroactive species between the bulk electrolyte and the underlying electrochemically active surface; when chosen properly, only the desired reactants and products can permeate the overlayer such that a single reaction is promoted on an otherwise unselective catalyst. Besides influencing selectivity, such coatings may also improve catalyst stability by providing mechanical support and by shielding the active catalyst from harmful catalyst poisons or undesirable side reactions, such as specific attack by chloride on a noble-metal component.^20^ Encapsulated electrocatalysts could hold great potential in solving selectivity problems, but they are still in a very early stage of development. To realize a properly functioning overlayer, its thickness and stability have to be optimal. The film should be just thick enough to block the undesired reaction without adversely affecting the mass transport of reactants and product species involved with the desired reaction. At the same time, the film itself must be chemically and physically stable, with strong adhesion to the underlying active electrocatalyst and/or support being essential to prevent physical delamination and ensure prolonged times of operation.

It was recently shown that manganese oxide (MnO*_x_*)-coated catalysts, which exhibit unusually high OER selectivity in acidic chloride electrolytes, effectively function as a perm-selective overlayer on the catalytically active IrO*_x_* material underneath.^29^ A MnO*_x_*-based overlayer is, however, not expected to be stable in acid for extended periods, although it can be made semistable within a precisely determined potential window.^30^ Other recent work demonstrates that nanometer-thick silicon oxide (SiO*_x_*) overlayers deposited by a photochemical deposition process onto planar Pt surfaces can perform a similar function.^31,32^ The SiO*_x_*|Pt electrodes were shown to selectively reject the transport of Cu^2+^ to the buried Pt catalyst, allowing the catalyst to remain active for the hydrogen evolution reaction in the presence of Cu^2+^, which normally inhibits the reaction via deposition onto Pt.^31^ SiO*_x_*|Pt was also shown to selectively reject chloride ion transport and thus effectively suppress the CER in favor of the OER in acidic conditions when used on flat, well-defined Pt surfaces.^32^ In contrast to MnO*_x_*, silicon oxides are expected to be thermodynamically stable in acid and at high potentials.^33,34^ A SiO*_x_* overlayer deposited onto known OER/CER catalysts could thus form a promising system for OER-selective seawater electrolysis in neutral or acidic media.

In this work, we explore the concept of using selectively permeable overlayers for enhancing selectivity toward the evolution of oxygen instead of chlorine in acidic chloride solutions. As a basis for this study, SiO*_x_* overlayers were deposited onto model Pt thin films, amorphous iridium oxide particles (IrO*_x_*), and IrO_2_-based high-surface-area catalysts on a Ti support (termed Ti-based anodes). Of these catalysts, Pt is known to be a highly active CER electrocatalyst, and as it has been studied repeatedly for SiO*_x_* deposition, this system forms a convenient reference point.^27,31,35,36^ IrO*_x_* and Ti-based anodes are representative of industrially relevant anodic materials used for catalyzing the OER in acidic and near-neutral water electrolysis.^37−39^ Thin-layered coatings composed of IrO*_x_* nanoparticles were included in this study to represent another well-defined model system for Ir-based electrocatalysts. Ir-based mixed metal oxide anodes supported on Ti, manufactured by Magneto Special Anodes (an Evoqua brand), were also studied; results involving these materials could in principle be directly translated to electrocatalysis under industrial conditions.^40^

## 2 Experimental Section

### 2.1 Electrochemical Procedures

KHSO_4_ and KCl (EMSURE) were purchased from Merck and used as received. The water used for all experiments was prepared by a Merck Millipore Milli-Q system (resistivity 18.2 MΩ cm, TOC < 5 p.p.b.).

All experiments were carried out at room temperature (∼20 °C). Electrochemical experiments were done using home-made two-compartment borosilicate glass cells with solution volumes of 100 mL. Before first-time use, all glassware was thoroughly cleaned by boiling in a 3:1 mixture of concentrated H_2_SO_4_ and HNO_3_. When not in use, all glassware was stored in a 0.5 M H_2_SO_4_ solution containing 1 g/L KMnO_4_. Before each experiment, glassware was thoroughly rinsed with water and then submerged in a dilute (∼0.01 M) solution of H_2_SO_4_ and H_2_O_2_ to remove all traces of KMnO_4_ and MnO_2_. The glassware was then rinsed three times with water and boiled in water. The rinsing-boiling procedure was repeated two more times.

An IviumStat potentiostat (Ivium Technologies) with the IviumSoft package was used during electrochemistry experiments. All experiments involving electrocatalytic chlorine and oxygen evolution were 95% iR-compensated. The solution resistance was measured with electrochemical impedance spectroscopy, by observing the absolute impedance in the high-frequency domain (100–10 KHz) corresponding to a zero-degree phase angle. Working solutions of 0.5 M KHSO_4_ were saturated with Ar (Linde, purity 6.0) before experiments. Solutions were bubbled with Ar gas during forced convection experiments, and Ar was used to blanket the solution in case of stationary conditions. The reference electrode for all RRDE experiments was a HydroFlex reversible hydrogen electrode (Gaskatel), separated from the main solution using a Luggin capillary, to fix the reference sensing point and to prevent mixed potentials at the reference due to dissolved Cl_2_ gas. The counter electrode was a Pt mesh, separated from the main solution by a coarse glass frit.

RRDE measurements were done with an MSR rotator and E6 ChangeDisk RRDE tips in a PEEK shroud (Pine Research). The Luggin tip connected to the reference electrode was aligned to the center of the RRDE electrode to minimize electrical cross-talk.^41,42^ Before chlorine or oxygen collection experiments, the Pt ring was electropolished by scanning from −0.10 to 1.70 V at 500 mV s^–1^ for 40 scans at 1500 RPM. The ring was kept at 0.95 V to selectively probe the CER in parallel with the OER, and at 0.40 V to probe the evolution of O_2_ in chloride-free electrolytes. Ring currents were corrected for constant background currents and product collection delay. the latter arises from the time needed for products formed on the disk to reach the ring.^43^

### 2.2 Electrode Preparation

#### 2.2.1 IrO*_x_*|GC

Commercial GC RDE disk inserts of 5 mm diameter were purchased from Pine Research. After hand-polishing the surface with diamond suspension and sonication in water, a thin IrO*_x_* layer was electroflocculated onto the GC surface from a hydrated IrO*_x_* colloid solution at acidic pH. Full details can be found elsewhere.^29^

#### 2.2.2 Pt|Ti|GC

Commercial GC RDE disk inserts of 5 mm diameter were purchased from Pine Research. A 2 nm layer of Ti (99.99%) and a 3 nm layer of Pt (99.99%) were sequentially deposited onto the GC surface at 0.2 A s^–1^ by electron beam evaporation without breaking vacuum and without substrate heating in an Angstrom EvoVac evaporator system, with a base pressure of 1.0 × 10^–7^ Torr. A commercial Pt disk from Pine Research served as SiO*_x_*-free reference material.

#### 2.2.3 Ti-supported Mixed Metal Oxides

Commercial Ti (grade 2) RDE disk inserts of 5 mm diameter were purchased from Pine Research. Two types of IrO_2_-based catalysts, a mixture of IrO_2_ and Ta_2_O_5_ and one of IrO_2_ and Pt, were prepared on these electrodes by Magneto Special Anodes (an Evoqua brand), using a thermal decomposition method.

#### 2.2.4 SiO*_x_* Deposition

Trimethylsiloxy-terminated polydimethylsiloxane (PDMS) dissolved in toluene was spin-coated onto the fixated disk samples with an acceleration 2400 rpm over 3 s, followed by a ramp to 4000 rpm over 30 s and maintained speed at 4000 rpm for 2 min following. The solvent was then evaporated by drying the electrodes in a vacuum oven at 90 °C for 60 min. To obtain SiO*_x_*, the final PDMS coating was chemically oxidized in a UV-ozone cleansing chamber for 2 h (UVOCS, T10X10/OES). The eventual SiO*_x_* film thicknesses were varied by changing the concentration of PDMS in the toluene solutions, and repeating the spin coating and drying procedure as necessary. For the SiO*_x_*|Pt|Ti|GC samples with a film thickness of 5 nm SiO*_x_*, a single spin coating step using a 5.3 mg/L solution of PDMS in toluene was chosen. Four SiO*_x_*|IrO*_x_*|GC samples were made with varied procedures to fabricate the SiO*_x_* overlayer: for sample 1, one drop of 10 mg/mL PDMS in toluene was used; for samples 2 and 3, two complete fabrication cycles were performed on each sample using one drop of 10 mg/mL PDMS in toluene for spin coating, and for sample 4, two complete fabrication cycles were performed using two drops of 10 mg/mL PDMS in toluene. The targeted SiO*_x_* overlayer thicknesses for these three procedures were 5, 10, and 20 nm, respectively. For the Ti-based anodes, three complete fabrication cycles were performed using 50 mg/L PDMS in toluene for spin coating. The targeted SiO*_x_* thickness on the Ti-based anodes was 10 nm. We note that the thickness of the overlayer may vary over the relatively rough surface of the Ir-based electrodes, especially regarding the mixed metal oxide samples on Ti supports. The SiO*_x_* thickness values for all samples are provided as estimates since the roughness associated with the substrates prevented reliable determination by ellipsometry.

### 2.3 Voltammetry Procedures during Electrocatalysis

All potentials in this work are reported versus the RHE scale. All currents were reported as densities per geometrical surface area. Normalization to the “real” catalyst surface area, which is an inherently difficult topic in electrocatalysis research,^20^ was not pursued; in this report, we were primarily interested in selectivity values (ratios of partial currents) and comparisons of activity before and after applying a SiO*_x_* overlayer. The electron beam-deposited Pt surfaces have a very low roughness (<1 nm) such that their active surface area is approximately equal to the geometrical one.^31^

#### 2.3.1 Pt

All Pt electrodes were pretreated before scanning by conditioning at 0.40 and 0.70 V for 10 and 3 s, respectively (see Figure S1), while rotating. This was to ensure that the surfaces were oxide-free and reproducible. Linear potential sweeps were performed immediately after on the Pt-based electrodes between 0.70 and 1.90 V, at 10, 20, and 50 mV s^–1^, under varying rotation rates. In-between experiments, the electrodes were kept at 0.05 V.

#### 2.3.2 IrO*_x_* and Ti-Based Anodes

Before initiating quantitative measurements, all Ir-based electrodes were scanned 20 times in a chloride-free electrolyte between 1.3 and 1.55 V (into the OER region) at 1500 RPM. This was done to ensure stable behavior during experiments by equilibrating the Ir centers (see below). Similar to Pt, a two-step potential-holding program preceded every catalytic cycle. The IrO*_x_* surfaces were conditioned for 10 and 3 s at 0.00 and 1.30 V, respectively; for the IrO_2_ + Ta_2_O_5_ catalyst, the procedure was 10 and 5 s at 0.00 and 1.30 V, and for IrO_2_ + Pt, it was 10 and 6 s at 0.05 and 1.30 V (see also Figure S1). After the pretreatment, all Ir-based catalysts were probed for OER and CER electrocatalysis between 1.30 and 1.55 V at 10 mV s^–1^ and 1500 RPM.

### 2.4 Scanning Electron Microscopy (SEM) and Energy-Dispersive X-ray Spectroscopy (EDS)

RDE inserts were carefully removed from the RRDE tip after electrochemical experiments and glued to a SEM specimen mount using conductive silver paint. The silver paint was dried for 3 h in air under reduced pressure. SEM micrographs were obtained using an Apreo S SEM setup (Thermo Scientific) equipped with a field emission electron source and EDS detector. Images were recorded in immersion mode using a through-the-lens detector, at a working distance of ∼4.0 mm, with 10 kV beam acceleration voltage and a beam current of 0.4 pA. EDS measurements were performed at the same beam voltage and current.

## 3 Results and Discussion

RRDE voltammetry was used to probe the kinetics and selectivity of the OER and CER on a variety of SiO*_x_*-modified catalysts. The use of an RRDE ensures well-defined mass transport conditions, which is important concerning accurate statements of selectivity where one reaction is fast and strongly dependent on diffusion. The use of interchangeable electrodes may cause some damage to the SiO*_x_* overlayer films, as will be described below; the method was nonetheless chosen as it offers an accurate and effective means of deconvoluting OER and CER current densities by employing a Pt ring to selectively reduce and quantify the Cl_2_ formed on the disk.^44^ The ring potential was fixed at 0.95 V vs RHE, which in the acidic media used in this work (pH ≈ 0.88) leads to diffusion-limited chlorine reduction and thus a quantitative correlation between the CER taking place on the disk and the ring current density *j*_R_, without interference from the oxygen reduction reaction. By correcting for the chlorine collection factor (*N*_Cl_2__), which represents the fraction of chlorine formed on the disk that is transported to and subsequently reduced at the ring, the geometric CER current density, *j*_CER_, can be calculated using the second term on the right-hand side of eq 3
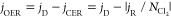
3

The remainder of the disk geometric current density *j*_D_ was then ascribed to OER (*j*_OER_, see eq 3), which is a reasonable approximation as long as capacitive scanning contributions to the disk current can be minimized. This was achieved using relatively slow scan rates and by averaging the values from forward and backward scans for Ir-related experiments. For the Pt experiments, a constant value was subtracted from the linear sweep voltammograms based on pseudo-capacitive charging seen around the onset of platinum oxide formation.

### 3.1 Pt Thin Films

The SiO*_x_*|Pt electrode is a convenient reference point for looking closely at parallel OER and CER and the effect of the SiO*_x_* overlayer. Pure Pt is not popular as an OER electrocatalyst for commercial electrolyzers due to its rather poor OER performance.^45^ At high potentials, the catalytic activity is also impacted by the formation of platinum oxides (PtO*_x_*), which will be discussed below. However, the excellent CER activity and poor OER activity of Pt create clear shifts in the onset potentials for these two reactions, making it easier to qualitatively interpret changes in the CER partial current density caused by the presence of the SiO*_x_* overlayers. Our groups have recently reported a separate, more focused investigation of the CER/OER behavior of SiO*_x_*|Pt electrodes.^32^ Here, we present RRDE characterization of performance of very similar electrodes and use them as a basis for evaluating the performance of SiO*_x_*-encapsulated electrodes composed of varied composition and/or structure. The RRDE setup allows more defined mass transport control and accurate selectivity measurement.

**Figure 1:**
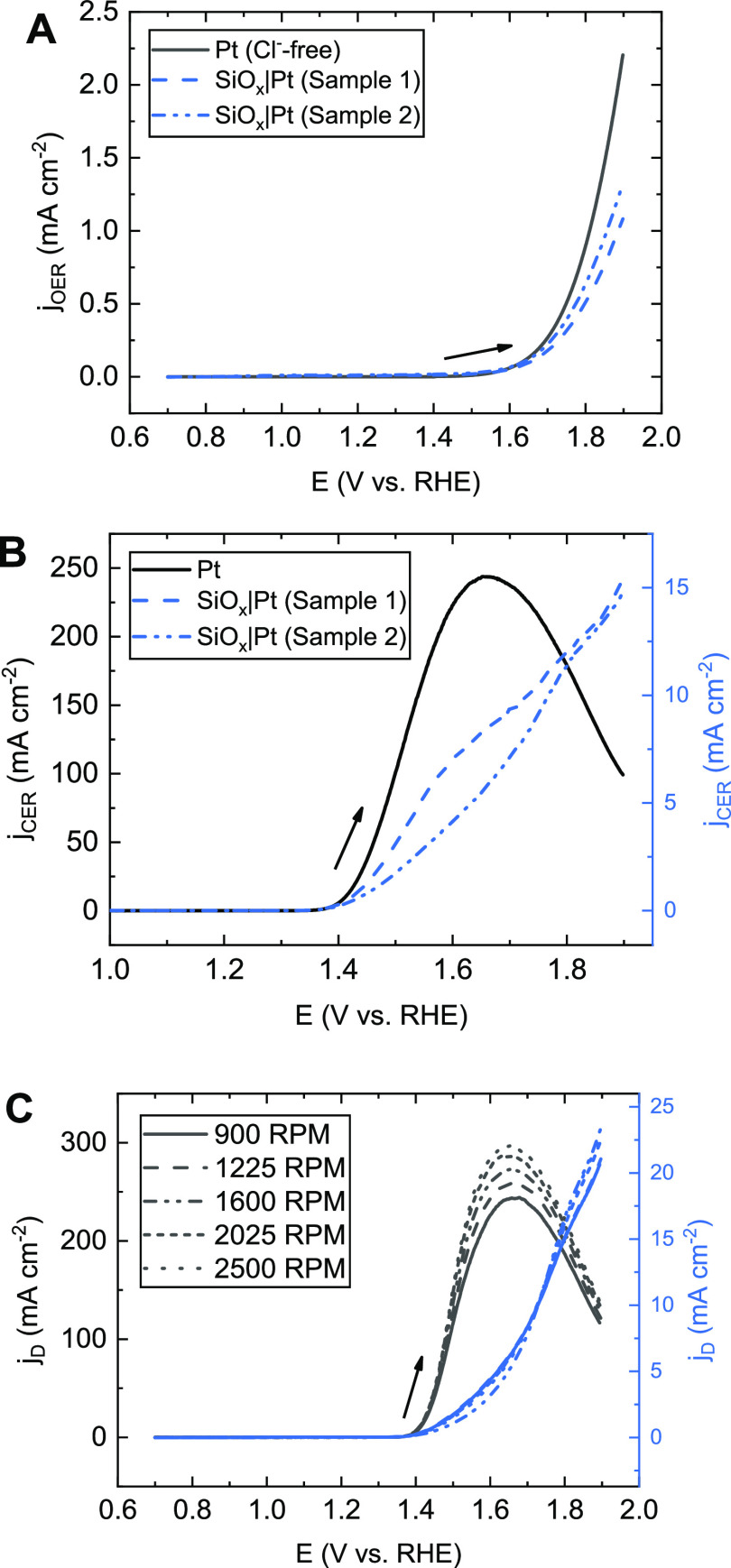
Effect of a SiO*_x_* overlayer on the electrocatalytic behavior of Pt in acidic chloride-containing media. (A) “Pure” OER activity on a Pt disk electrode (black) and two SiO*_x_*|Pt|Ti|GC electrodes with a 5 nm SiO*_x_* overlayer (blue), in 0.5 M KHSO_4_ (chloride-free conditions). Rotation rate 1600 RPM, LSVs recorded at 10 mV s^–1^. (B) Current densities of the CER in 0.5 M KHSO_4_ + 0.6 M KCl, on Pt (black) and on the SiO*_x_*-coated Pt samples (blue). Note the difference in scale. j_CER_ was derived from ring currents as described in eq 3. (C) Measured disk current density versus rotation rate on Pt (black) compared to a 5 nm SiO*_x_*|Pt|GC electrode (blue), in 0.6 M chloride, recorded at 20 mV s^–1^. Arrows indicate scan direction.

Figure 1 shows some typical results of parallel OER and CER on bare and SiO*_x_*-covered Pt surfaces. LSV curves measured in a chloride-free electrolyte, for which all current can be attributed to the OER, are shown in Figure 1A, revealing that the OER activity is only moderately impacted by the overlayer. It was verified that the OER still occurs on the SiO*_x_*-encapsulated catalyst using the RRDE with the Pt ring fixed at 0.40 V vs RHE in chloride-free conditions, showing that O_2_ can traverse the overlayer to be detected on the ring (Figure S4). Figure 1B shows LSVs involving parallel oxygen and chlorine evolution, which were measured in the presence of 0.6 M KCl, roughly the average chloride concentration of natural seawater.^46^ On an unmodified Pt electrode (black trace in Figure 1B), chlorine evolution has a clear onset around 1.37 V vs RHE, after which the rate goes through a maximum and declines; the latter can be ascribed to inhibiting effects from PtO*_x_* formation at high potentials.^47^ It has been previously reported that PtO_x_ already interferes with the CER around its onset;^48−50^ the peak CER current seen in Figure 1B is around 16% of the value predicted by the Levich equation, meaning that the maximum is significantly lower than would be expected from diffusion limitations. In the presence of a SiO*_x_* overlayer (blue traces), the CER activity is strongly inhibited and decreases roughly 20-fold relative to the bare Pt electrode. The rotation rate dependence of the disk current density in the chloride-containing electrolyte is shown in Figure 1C for both electrodes. This experiment is useful for uncovering the relative importance of Cl^–^ transport across the diffusion boundary layer in the bulk liquid electrolyte to that across the SiO*_x_* overlayer, since only the thickness of the former will change with rotation rate. Contrary to bare Pt, there is very little dependence of disk current on the chloride mass transport to the SiO*_x_*|Pt buried interface, suggesting that the observed potential-current response (which is largely from chlorine evolution) of that sample is dominated by chloride transport through the SiO*_x_* overlayer. This furthermore indicates that the concentration overpotentials and associated concentration gradients across the bulk diffusion boundary layer are very small compared to those across the SiO*_x_* diffusion barrier under these conditions.

We note that the CER rates on SiO*_x_*|Pt|Ti|GC in Figure 1B,C do not show a maximum current density as seen for bare Pt, suggesting that the overlayer changes how PtO*_x_* forms during the scan. The voltammetric characterizations in Figure S2 illustrate that in the presence of the SiO*_x_* overlayer, the onset of PtO_x_ formation is similar, but more oxide appears to be formed on SiO*_x_*|Pt relative to the SiO*_x_*-free sample, after normalizing the oxide reduction peaks to the electrochemical surface area using the hydrogen desorption region (Figure S3). This is likely because SiO*_x_* overlayers have been shown to reject the transport of the bisulfate anions in the aqueous KHSO_4_ electrolyte that adsorb to Pt surfaces and inhibit Pt oxidation.^32^ SEM micrographs (Figure S15) suggest that the surface of the as-made electrode is homogeneously covered by SiO*_x_*. Neither Si associated with the SiO*_x_* overlayer or Pt and Ti from the electrode and adhesion layer could be identified in EDS analysis due to the limited interaction between the electron beam and these ultrathin layers.

Even though the suppressive effect of SiO*_x_* on the CER is large, it is not quite as large as measured during a similar study by us using stationary, ideally flat electrodes, where the residual CER activity over the same potential range was less than 1%.^32^ The higher residual CER activity observed in this work suggests that the overlayer integrity was compromised to some extent, possibly by mechanical stress. This could be caused by hydrodynamic forces under rotation, or by those that arise from pressing the disk electrode into the RRDE assembly, during which the disk extremities are unavoidably subjected to force. The latter could explain the CER activity in Figure 1 since we could not find signs of defects while broadly surveying the covered surfaces with SEM. The absence of clear rotation rate dependence in Figure 1C suggests that chloride diffusion relating to the CER activity is still somehow hindered.

**Figure 2:**
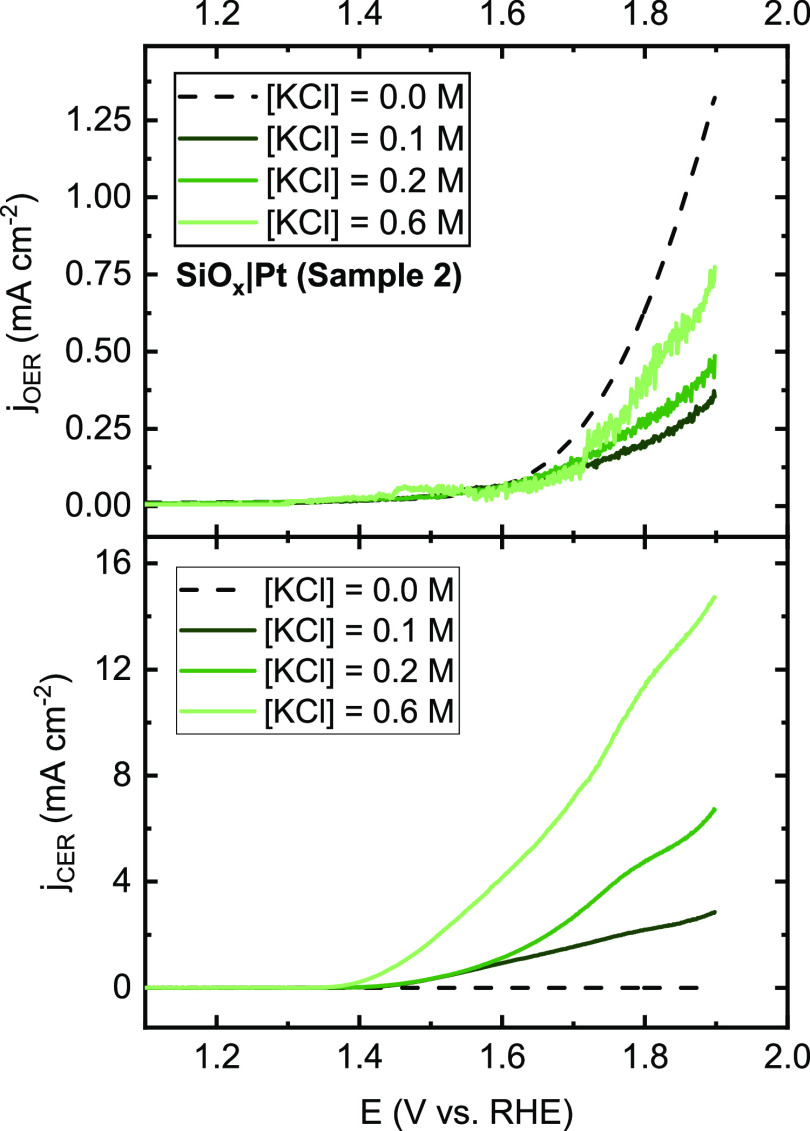
Partial current densities for the OER (top) and CER (bottom) as a function of chloride concentration on a 5 nm SiO*_x_*|Pt|Ti|GC electrode in 0.5 M KHSO_4_ with varying concentrations of KCl. Rotation rate 1600 RPM, LSVs recorded at 10 mV s^–1^.

The variation of the OER and CER activity as a function of the chloride concentration on a SiO*_x_*|Pt|Ti|GC sample is shown in Figure 2. We found that the CER rate on the bare Pt surface (Figure S11) displays an approximately linear response to the concentration, indicating that the chloride reaction order is close to 1. In Figure 2, however, it can be seen that the SiO*_x_*-coated samples have different, more complex concentration dependencies. Figure 2 (top) shows that the OER activity at the buried interface is slightly suppressed by the addition of chloride, but otherwise not strongly dependent on the chloride concentration.

On bare Pt, the derivation of OER currents in parallel with the CER was unfortunately not possible, due to the high rates of chlorine evolution on such surfaces; these high rates led to macroscopic gas bubbles that lodged at the interspace between the disk and the ring and led to severe distortion of the ring response, as reported previously.^51^ The CER currents on the SiO*_x_*|Pt|Ti|GC electrodes were much lower so that it was still possible to use the ring-disk approach in highly concentrated chloride solutions, although the ring response was still slightly erratic; the apparent value of *N*_Cl_2__ varied somewhat (±10%) depending on the sample. This is possibly a result of hindered transport of Cl_2_ across the SiO*_x_* film and leads to the somewhat erratic behavior of derived OER currents seen in Figure 2 (top). To minimize this source of error, *N*_Cl_2__ was calibrated for each experiment by comparing the disk current to the ring response while evolving chlorine in the potential region 1.50–1.55 V, where the CER is the sole reaction taking place.

Selectivity between the OER and CER is of central importance for direct seawater electrolysis. In this paper, the molar selectivity toward the CER was calculated as the ratio of moles Cl_2_ formed versus total moles formed
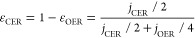
4

Based on the results of Figures 1 and 2, it is clear that the SiO*_x_* overlayer strongly affects the OER vs CER selectivity on Pt, but a quantitative description of the observed behavior becomes complicated as multiple effects are involved. On the one hand, the overlayer strongly inhibits the CER; on the other hand, it also seems to inhibit PtO_x_ formation, which can favor chlorine evolution. The SiO*_x_* overlayer also leads to some inhibition of the OER activity compared with the bare Pt surface (Figure 1A), which further increases ε_CER_. Full selectivity data are shown in Figure S5; one can observe that the bare Pt surface can show a slight enhancement of the OER selectivity at high potentials (from 6 to 7%), due to the strong suppression of the CER by platinum oxides. Nonetheless, the most important finding is that the SiO*_x_* overlayer on Pt greatly impairs the CER, while still allowing the OER to occur.

### 3.2 IrO*_x_* Nanoparticles

Thin layers of electroflocculated, hydrous IrO*_x_* have shown significant activity for the OER and CER.^44,52−54^ We therefore deposited SiO*_x_* onto this material and tested whether the CER could be selectively suppressed. The targeted SiO*_x_* thickness for the IrO*_x_* samples was 5–20 nm, generally much higher than that used for the smooth Pt thin film samples. Higher target thicknesses were chosen because the IrO*_x_* film has roughness features in the order of 10–100 nm (Figure S19), substantially larger than the rms roughness of <1 nm reported for the SiO*_x_*|Pt thin films. Previously, we have found that the UV-ozone deposition process results in thinner SiO*_x_* overlayers on the top of particles and thicker layers deposited on the substrate in the areas in between the particles,^55^ and similarly expect that the actual SiO*_x_* overlayer thickness varies significantly on the four SiO*_x_*|IrO*_x_* samples investigated here alongside a SiO*_x_*-free control sample.

**Figure 3:**
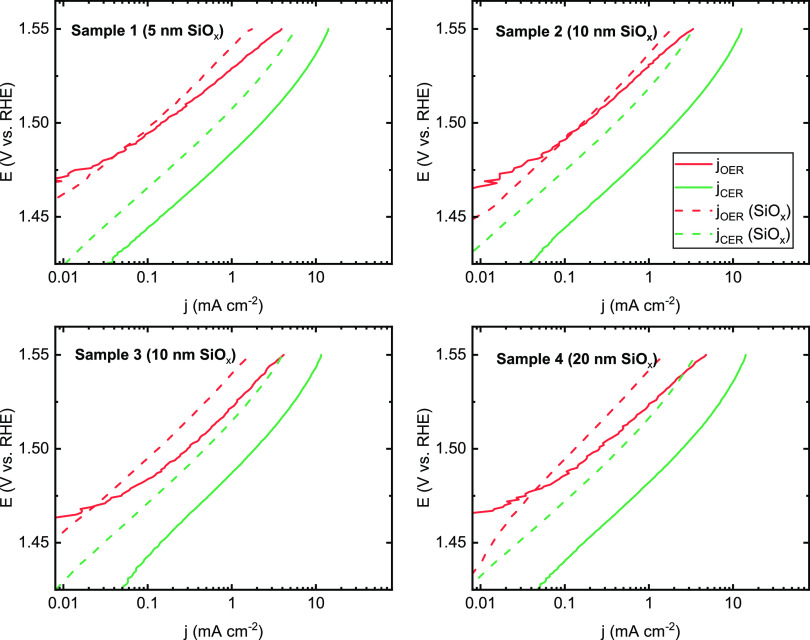
Tafel plots for parallel OER and CER on SiO*_x_*|IrO*_x_* samples of varying SiO*_x_* overlayer thicknesses, before and after SiO*_x_* deposition, in 0.5 M KHSO_4_ + 30 mM KCl. Rotation rate 1500 RPM.

Figure 3 shows a comparison of Tafel curves based on the OER and CER partial current densities, derived from RRDE measurements using eq 3, in a chloride concentration of 30 mM as a test case for comparing the sample activities before and after SiO*_x_* deposition. The first trace of evolved oxygen appeared between 1.45 and 1.46 V on these samples, regardless of whether SiO*_x_* was present (Figure S7). In the presence of a SiO*_x_* layer, the OER activity of the IrO*_x_* samples decreases moderately. The Tafel lines of the OER and CER were both shifted to higher potentials; this lowering of activity is usually more severe for the CER, but all IrO*_x_* samples still showed considerable CER activity after the SiO*_x_* coating. This is contrary to the results for Pt described in Section 3.1, where the SiO*_x_* overlayer on Pt decreased the CER activity to less than a few % relative to the uncoated Pt reference sample. This means that either the IrO*_x_* is incompletely covered by the films, or the films are somehow not as effective at suppressing CER when IrO*_x_* is the underlying substrate. Consistent with this latter explanation, Beatty et al. have reported that the substrate composition can strongly impact the permeability of SiO*_x_* overlayers toward proton and Cu^2+^ transport.^36^

**Figure 4:**
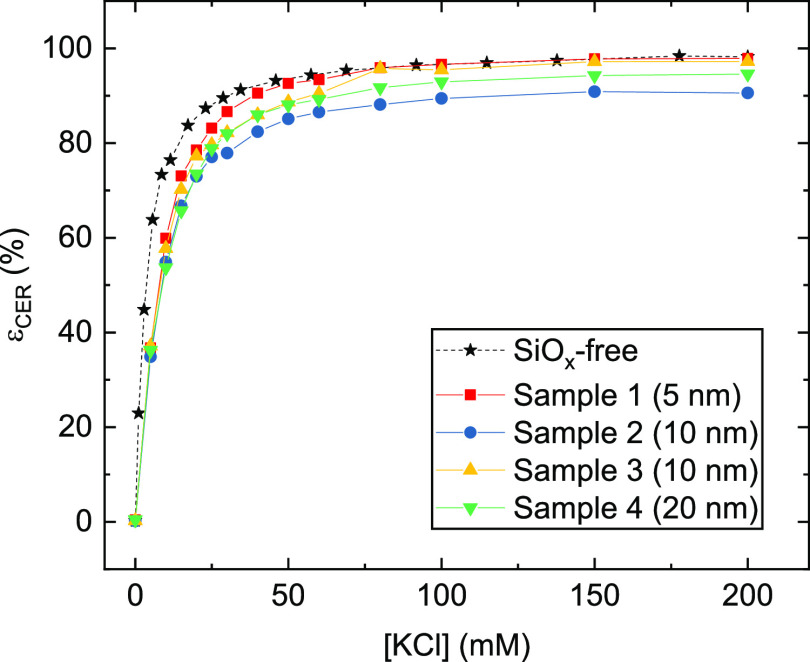
Molar selectivity toward the CER as a function of chloride concentration for SiO*_x_*|IrO*_x_* samples with varied target thickness of the SiO*_x_* overlayer. Values of ε_CER_ calculated according to eq 4. Rotation rate, 1500 RPM.

The CER selectivity (ε_CER_) of the SiO*_x_*|IrO*_x_*|GC electrodes was measured as a function of chloride concentration, where the upper limit of 200 mM KCl is reasonably close to the actual chloride concentration in seawater (Figure 4). Although the samples all show some reduction in CER selectivity compared to the SiO*_x_*-free reference sample, there is no clear correlation between the selectivity and the target SiO*_x_* thickness.

To look more closely into the effect of the SiO*_x_* overlayer, we focused on the kinetics of both reactions by looking at experimental Tafel slopes and chloride reaction orders  (Figures S12 and S14). Although caution is advised when trying to directly interpret either of these quantities in relation to the “true” underlying reaction mechanism, it is expected that they change significantly when the SiO*_x_* overlayer exerts an influence, especially so for the CER. If diffusion is the limiting step, values of  for the CER should be 1 and the Tafel slope should approach infinity. These values should be attained when the potential-dependent CER rate determined by reaction kinetics exceeds the rate of mass transport. When comparing  on the bare IrO*_x_*|GC sample and SiO*_x_*|IrO*_x_*|GC (Section 2 in the Supporting Information), there is no obvious change in the values as a function of potential. We do note that CER Tafel curves on all SiO*_x_*-coated samples have slightly higher slopes around the positive potential limit of 1.54 V; they are 65–70 mV/dec, compared to ∼55 mV/dec in the reference. This could be associated with concentration overpotentials from suppressed chloride transport through the overlayer, but only to a minor extent. As the OER activity is also slightly suppressed, we investigated the voltammetric characterizations of the samples before and after SiO*_x_* deposition (Figure S6). The presence of SiO*_x_* seems to suppress the semireversible peak observed around 0.94 V, which is ascribed to a redox transition between Ir^3+^ and Ir^4+^.^56−58^ Suppression of the peak shows that the overlayer affects the redox states in the IrO*_x_* film and that the overall reaction kinetics could change because the catalytic behavior of IrO*_x_* intimately depends on the redox state of the Ir centers.^59−61^ However, we found (Section 2 in the Supporting Information) that linear Tafel slopes of the OER in all samples ranged between 40 and 50 mV/dec, and that likewise, the OER activity was little affected by chloride (Figure S13), irrespective of the presence of the SiO*_x_* overlayer. Both observations indicate that the OER mechanism remains the same after applying the SiO*_x_* coating. Therefore, the SiO*_x_* likely does not affect the reactivity of the IrO*_x_* underlayer. One possible explanation for the similar Tafel slopes and high CER activities for both SiO*_x_*-encapsulated and bare IrO*_x_* electrodes is that the SiO*_x_* overlayer is incomplete, or locally delaminates during electrode conditioning and/or gas evolution.

SEM and EDS were used to analyze changes in the morphology and composition of the samples; Figure S17 shows a typical EDS spectrum of an SiO*_x_*|IrO*_x_*|GC electrode with target SiO*_x_* thickness of 10 nm taken after electrolysis experiments. Besides Si, Ir, and O, large amounts of C were always detected due to the bulk GC electrode support. Cl was also usually seen in low amounts, along with K and S; these correspond to traces of electrolyte remaining on the electrode surface. Part of the Cl fraction is likely bound to Ir as a result of incomplete hydrolysis of the chloroiridate precursor.^29^

**Figure 5:**
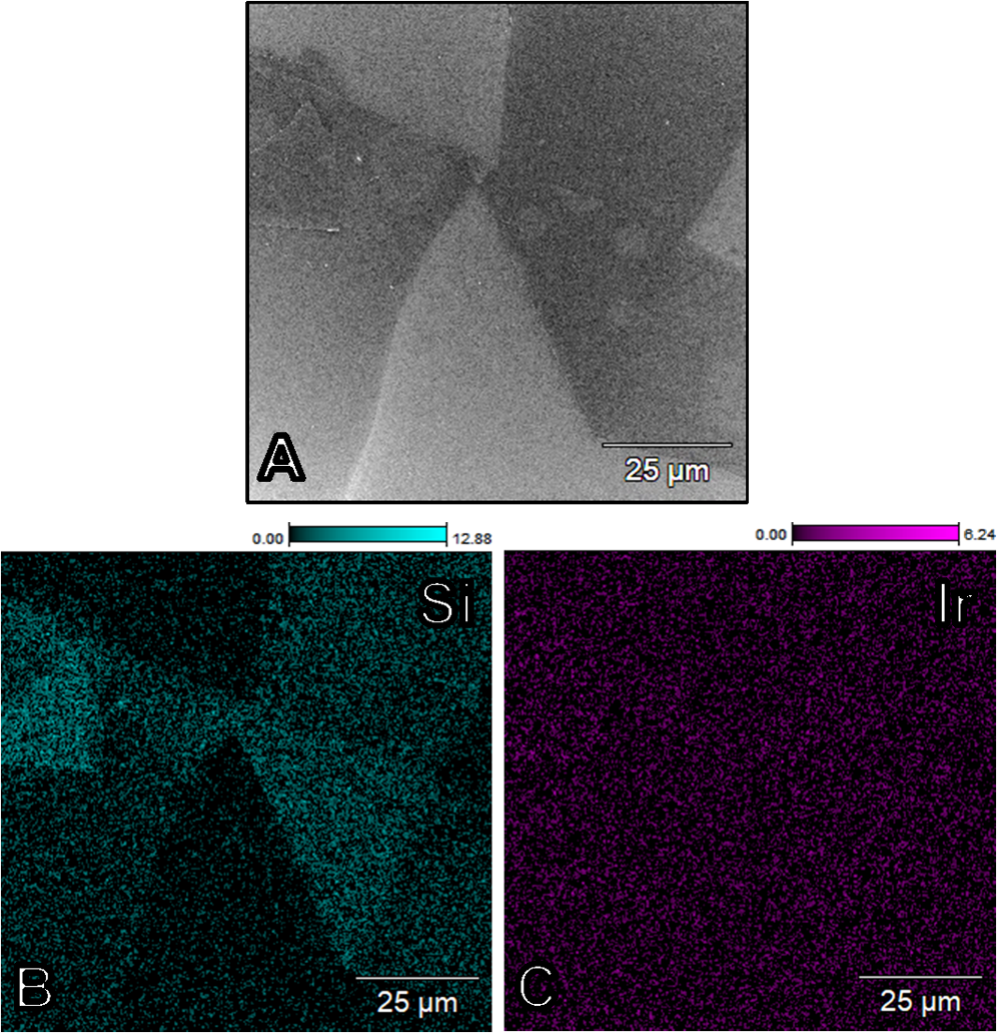
SEM micrograph and EDS analysis of a SiO*_x_*|IrO*_x_*|GC electrode surface, after extensive OER + CER electrocatalysis under forced convection conditions. In the electron image (A), folded sheets of SiO*_x_* are visible on top of the IrO*_x_* layer. Color images (B, C) show the corresponding elemental mapping of Si and Ir. Scale bars show the amounts of the elements, as the atomic percentage of total (which included O and C from the GC substrate, see Figure S18).

An example micrograph of a SiO*_x_*|IrO*_x_*|GC surface after extensive electrochemical measurements is shown in Figure 5, together with corresponding elemental maps of Si and Ir (see Section 3 in the Supporting Information for additional micrographs). In the SEM image provided in Figure 5A, the surface of the sample appears patchy, with clear delineation between dark and bright areas. These features contrast sharply with what is typically observed for the “bare” IrO*_x_*|GC samples (Figure S16), as well as other areas of the SiO*_x_*|IrO*_x_*|GC surface (see the comparison in Figure S20), which have a relatively featureless appearance in the micrographs. EDS analysis (Figure 5B) shows that the SiO*_x_* is present across the entire image but not distributed evenly, with the dark gray areas from the SEM image corresponding to areas of higher Si EDS intensity. The variations in Si EDS intensity in Figure 5B correlate strongly with the contours seen in the electron map in Figure 5A, with intensities staying relatively constant across different patches. This observation suggests that the SiO*_x_* overlayer experienced thinning and/or partial delamination in some places on the sample, where the latter phenomena may have coincided with the disconnected overlayer folding back on top of itself to give multilayer regions with higher Si EDS signal intensity. Si was found globally across the electrode surface, including areas where the IrO*_x_* layer was interrupted or where local clusters of IrO*_x_* nanoparticles were formed (Figures S21 and S22).

High-resolution micrographs provided in Figure S19 illustrate the morphology of these clusters. Rugged surface structures are difficult to cover properly via spin coating so that the thickness or presence of SiO*_x_* layer around these clusters could be nonuniform compared to the SiO*_x_* deposited on the smooth Pt thin films; it is not possible to register such imperfections with EDS, which is inherently limited to resolutions of around a micrometer. Nonetheless, we could not find indications that large areas of the electrode were completely devoid of SiO*_x_*. The previously discussed CER activity then probably originates from nano- or microscopic areas where the electrocatalytic activity is high and the SiO*_x_* overlayer is imperfect, or damaged by local instances of vigorous gas evolution at the buried interface (see Figures S23 and S24). Supporting this hypothesis, recent work by Stinson et al.^62^ has used scanning electrochemical microscopy (SECM) to show that low densities of microscopic defects in thin SiO*_x_* layers encapsulating Pt thin films can significantly decrease electrode selectivity toward a reaction occurring at the buried interface compared to a reaction which only occurs at the defects. Overall, characterization of the SiO*_x_*-encapsulated electrodes studied in this work suggests that overlayer adhesion (and therefore the occurrence of overlayer defects) can depend strongly on the morphology and/or composition of the substrate material, and that care must be taken when translating a functional overlayer design to a different catalyst.

### 3.3 Ti-supported Mixed Metal Oxides

The Ti-supported anodes are composed of thick layers of electrocatalytic metal oxides deposited on Ti RDE disks using the same method employed for fabricating large surface area anodes that are sold commercially by Magneto Special Anodes (an Evoqua brand). We tested a sample consisting of a Ti substrate coated with a mixture of IrO_2_ and Ta_2_O_5_ (termed IrTa|Ti), as well as two samples of an IrO_2_ anode containing Pt (termed IrPt|Ti). The OER and CER activity of these samples was evaluated before and after SiO*_x_* deposition on the same samples. As this type of electrode commonly has micrometer-sized roughness,^63−65^ larger concentrations of SiO*_x_* precursor were used during spin coating deposition to account for the increase in active surface area.

**Figure 6:**
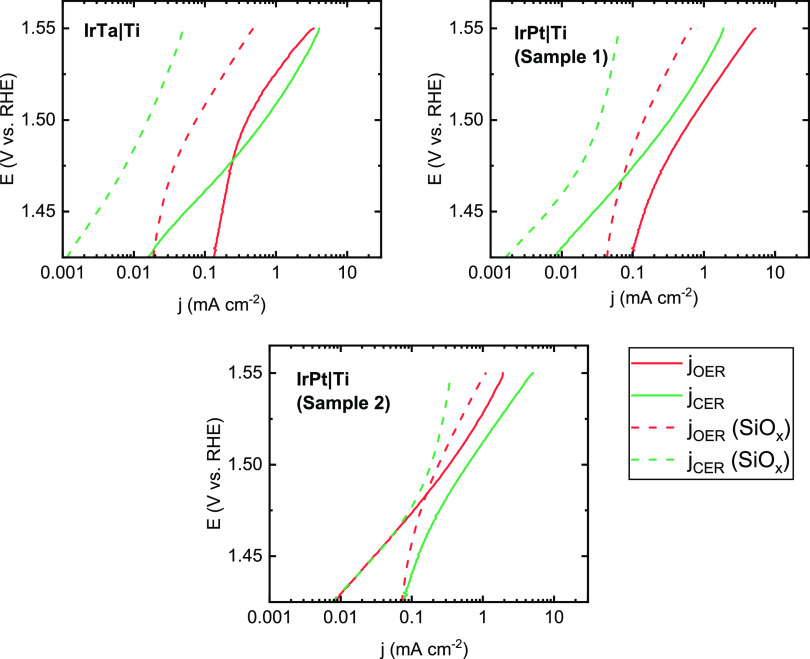
Tafel plots for parallel OER and CER on Ti-based anodes, before and after SiO*_x_* deposition, in 0.5 M KHSO_4_ + 30 mM KCl. Rotation rate 1600 RPM.

Figure 6 shows Tafel curves of the Ti-based anode samples in 30 mM KCl, before and after depositing SiO*_x_*. The presence of the SiO*_x_* overlayer results in a significant decrease in overall activity for all Ti-based anode samples compared to the bare control samples. Total current densities decreased by roughly 80–90%, compared to 50–60% for the IrO*_x_* samples. This is most likely caused by the relatively thick overlayers deposited on the Ti-based anodes, which may hinder mass transport as the thickness increases.^27^ Evidence for this is a decrease in redox features in the voltammetric characterizations of the samples after the coating (Figure S8). Besides lower activity, there is also significant suppression of the CER in favor of the OER, which is a favorable finding; the effect is much stronger than seen on the IrO*_x_*|GC samples discussed in Section 3.2.

**Figure 7:**
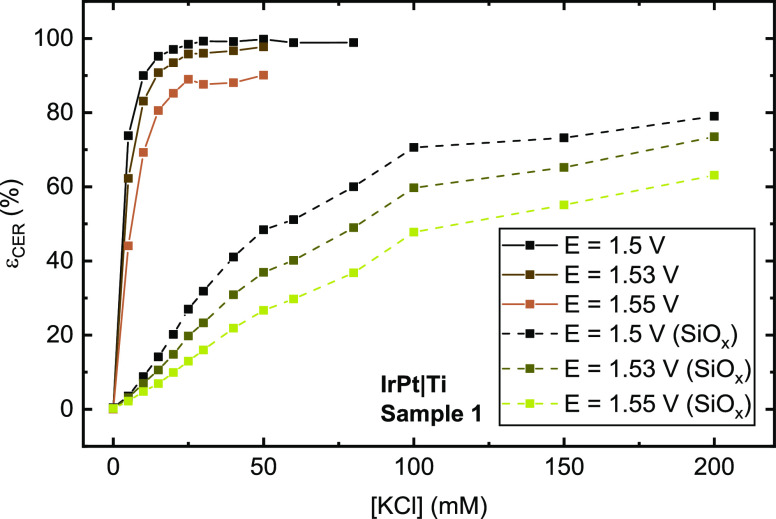
Molar selectivity toward the CER as a function of chloride concentration for an IrPt|Ti sample. Values obtained on the unencapsulated anode (solid, brown lines) are compared to those in the presence of the SiO*_x_* overlayer (beige, dotted lines). Rotation rate 1500 RPM.

To evaluate selectivity, ε_CER_ was determined as a function of chloride concentration for the bare and SiO*_x_*-encapsulated Ti-based anodes using the same procedure already described in Section 3.1. The results in Figure 7 reveal that the SiO*_x_* overlayer has a substantial effect on the CER selectivity, greatly favoring the evolution of oxygen relative to chlorine at all chloride concentrations, but especially at concentrations below 30 mM. A similar trend was seen on the other Ti-based anode samples (see Figures S9 and S10).

**Figure 8:**
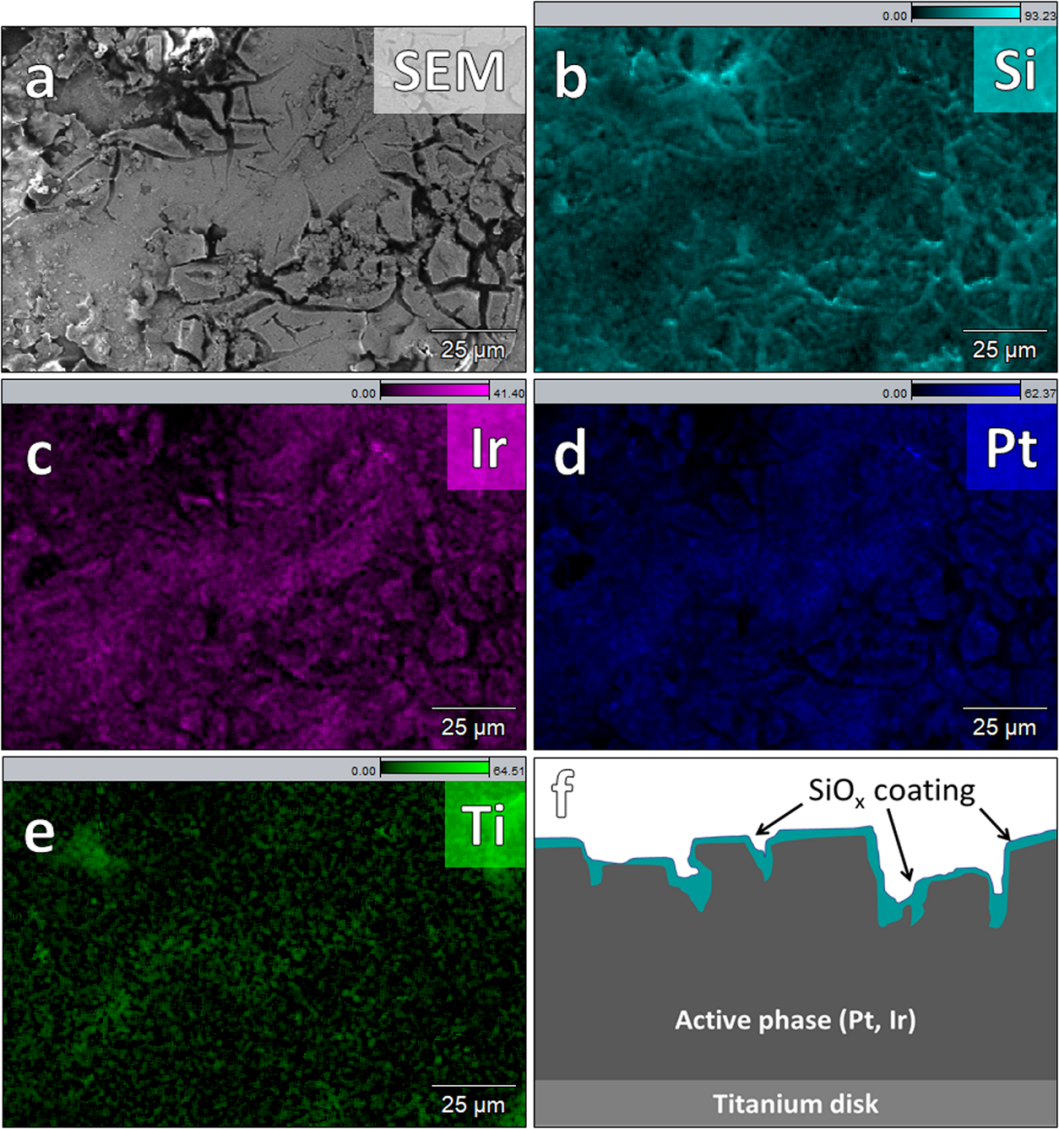
SEM micrograph and EDS mapping of an IrPt|Ti electrode surface after extensive OER + CER experiments under rotation. The electron image is shown in grayscale, and colored images show the elemental mapping of Si, Ir, and Pt. Scale bars show the relative amounts of the elements, as an atomic percentage of total (see Figure S25 for extra data on C and O).

The higher OER selectivity for the Ti-based anode samples relative to the IrO*_x_* samples might be partially due to better continuity of the thicker SiO*_x_* overlayer, but could also be related to differences in SiO*_x_* overlayer durability, induced by changes in the underlying morphology. As seen in the SEM images provided in Figure 8A, the Ti-based anodes exhibit roughness features that are substantially larger than those observed on the IrO*_x_* thin film electrodes. SEM/EDS analysis of an IrPt sample in Figure 8B shows that Si has accumulated in the micrometer-sized cracks of the mixed metal oxides. This accumulation must have occurred during the spin coating phase, where the PDMS solution infiltrated the catalyst cracks. As the distribution of Si around the cracks is not strongly coupled to the carbon signal (see Figure S25A), it is probably present as the oxide and not its PDMS precursor. It suggests that despite being isolated within the cracks, the excess PDMS was still successfully oxidized during the plasma treatment or oxidized later during polarization experiments. Regardless of the exact state of the SiO*_x_* overlayers, we hypothesize that the accumulation of the SiO*_x_* material within those cracks could serve as anchor points that help to enhance the adhesion of the overlayer to the electrode surface and maintain high ε_CER_ throughout the course of the RRDE experiments. See Figure 8F for an illustration.

**Figure 9:**
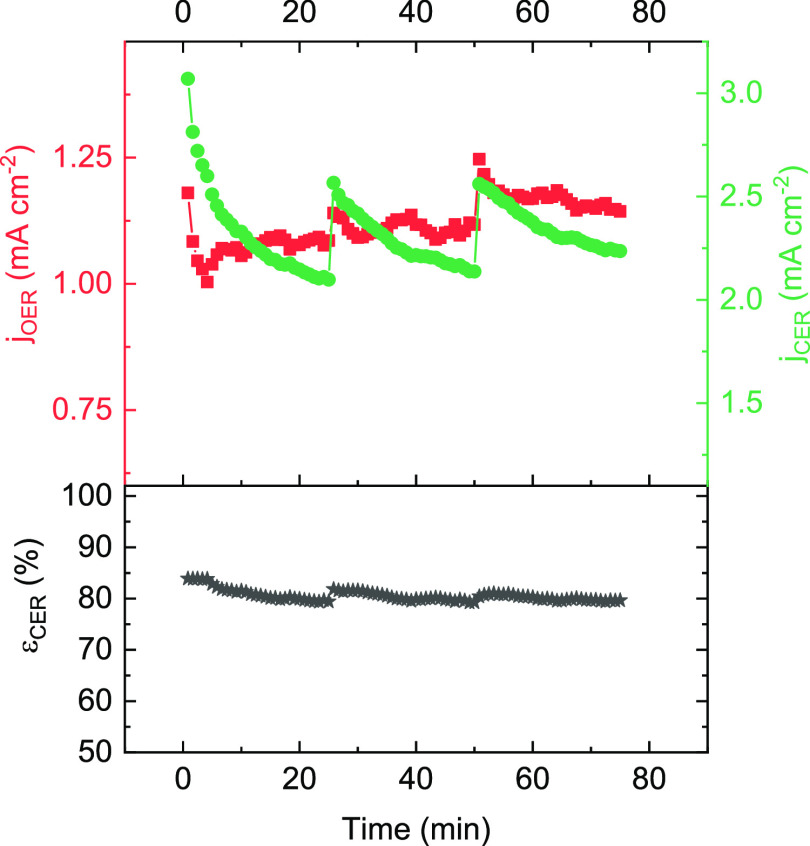
Accelerated lifetime test of IrPt|Ti sample 1 by repeatedly scanning between 1.3 and 1.55 V, in 0.5 M KHSO_4_ + 200 mM KCl. Rotation rate 1500 RPM, OER and CER currents gathered at a potential of 1.55 V.

Finally, we also probed the stability of the SiO*_x_* overlayer, which is of vital importance when the electrodes are implemented for industrial purposes. The Ti-based anodes were developed for large-scale electrolysis, and they are designed for up to several years of stable operation, depending on the application. As an accelerated stability test, a IrPt|Ti electrode was scanned repeatedly in and out of the mixed OER + CER region, between 1.30 and 1.55 V vs RHE, under rotation at 1500 RPM in 200 mM KCl. These conditions were chosen as catalyst stability in oxygen and chlorine electrocatalysis is usually the lowest under potentiodynamic conditions.^66−68^ A total of 100 cycles were applied over three intervals, amounting to roughly 75 min, or 25 min per interval. The catalytic activity and CER selectivity were monitored over time, with the results provided in Figure 9 (top and bottom), respectively. The use of intervals in the experiment was necessary to monitor and correct for detrimental gas bubble formation at the ring-disk interface in between the experiment.

Both the OER and CER activities (Figure 9, top) show a relatively complex pattern, where within a session of 25 min, the activity is initially high and then shows a decline. This behavior can be explained by the dynamic redox nature of IrO_2_-based materials. The electrocatalyst is likely in a partially reduced state at the start of a session, leading to a higher activity, but as the material is transiently reoxidized during anodic polarization, the activity declines.^69^ Importantly, the CER selectivity stayed very close to a constant value of ∼80% throughout the entire experiment. The steady selectivity suggests that the overlayer integrity is well preserved during prolonged potentiodynamic electrolysis. All in all, the results in this section show that a SiO*_x_* overlayer is capable of significantly increasing the OER selectivity of an industrially relevant catalyst; however, more research is needed to further improve this selectivity and reduce the negative impact on catalyst activity toward the desirable OER.

## 4 Conclusions

The work described in this paper shows that SiO*_x_* encapsulation is a promising approach to enhancing selective oxygen evolution on high-surface-area, industrially relevant anodes in acidic, chloride-containing electrolytes. RRDE studies of SiO*_x_*-encapsulated Pt thin film electrodes show that this type of barrier is capable of suppressing the rate at which chloride ions reach the catalytic buried interface while allowing oxygen evolution to still take place. The application of the same overlayer to Ir-based catalysts, which are much more representative of anodic materials used in commercial electrolyzers, led to varying success. On nanoparticulate, amorphous IrO*_x_*, the SiO*_x_* overlayers were not effective enough to lower the CER selectivity to satisfactory values. It is likely that the SiO*_x_* film integrity on this type of substrate was compromised, as the residual CER activity behaved kinetically very similarly to that observed on unmodified IrO*_x_* surfaces. The overlayer failure could be due to activity hotspots on the surface that lead to intense gas evolution and delamination at the buried interface, or generally insufficient interaction of the overlayer with the catalyst. Application of an extra thick SiO*_x_* overlayer to Ti-supported mixed metal oxides led to a significant increase in OER selectivity and also a notable suppression in the OER and CER current densities at a given potential.

It must be stressed that the SiO*_x_* deposition method can and should be further optimized to maximize adhesion and selectivity while avoiding substantial decreases in the OER current density. Although spin coating of a sol–gel precursor layer was employed in this paper, dip coating, spray coating, or alternative wet chemical methods such as condensed layer deposition^70^ are expected to be better suited for achieving more conformal coatings on rough surfaces. It must also be stressed that other oxide materials besides SiO*_x_* could be employed as perm-selective overlayers, such as MoO*_x_*, VO*_x_*, or (at high anodic polarization) CeO*_x_*.^71−73^ Alternatively, polymer modification or thin-membrane approaches might be used.^74^ In any case, this study suggests that the morphology of the underlayer and its interaction with the overlayer are highly important in making the buried interface stable and effective. Further research into membrane-coated electrocatalysts may be a very promising pathway toward the realization of selective seawater electrolysis.

## Supporting Information Available

Supplementary voltammetry data; kinetic OER and CER data; and supplementary SEM/EDS data (PDF)
