# Supplementary material for: Probing the Effects of Electrode Composition and Morphology on the Effectiveness of Silicon Oxide Overlayers to Enhance Selective Oxygen Evolution in the Presence of Chloride Ions
Source: J Phys Chem C Nanomater Interfaces. 2022 Nov 22;126(48):20314–25. doi: 10.1021/acs.jpcc.2c07116 (PMC9743210; doi:10.1021/acs.jpcc.2c07116)
Supplement: Supplementary file 1 — jp2c07116_si_001.pdf [file jp2c07116_si_001.pdf]

**Supporting Information**

# Probing the Effects of Electrode Composition and Morphology on the Effectiveness of Silicon Oxide Overlayers to Enhance Selective Oxygen Evolution in the Presence of Chloride Ions

Johannes G. Vos,[a,b] Amar A. Bhardwaj,[c] Adriaan W. Jeremiasse,[b] Daniel V. Esposito,[c] and Marc T.M. Koper[a*]

[a] Leiden Institute of Chemistry, Leiden University, PO Box 9502, 2300 RA Leiden, The Netherlands
[b] Magneto Special Anodes (an Evoqua brand), Calandstraat 109, 3125 BA Schiedam, The Netherlands
[c] Department of Chemical Engineering, Columbia Electrochemical Energy Center, Lenfest Center for Sustainable Energy, Columbia University in the City of New York, 500 W. 120th Street, New York, New York 10027, United States
* Corresponding author, e-mail address: m.koper@lic.leidenuniv.nl

# Contents

1. Supplementary voltammetry data ........ S3
2. Kinetic OER and CER data ........ S9
3. Supplementary SEM/EDS data........ S11

## 1. Supplementary voltammetry data

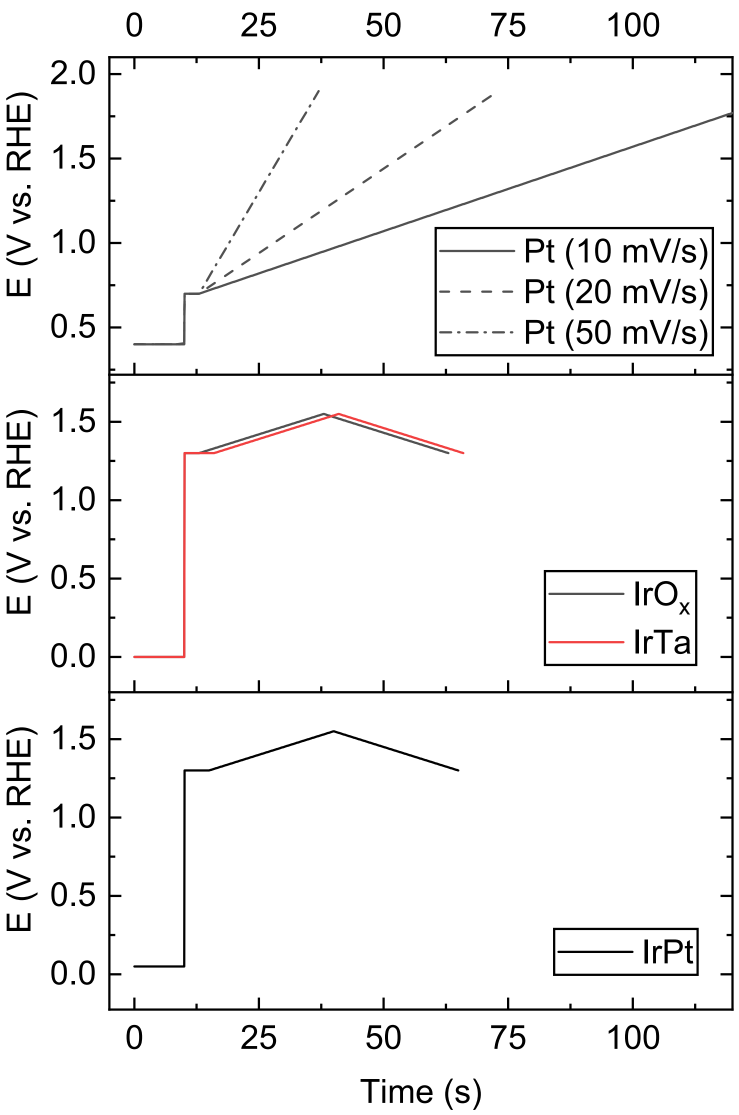


*Figure S 1: E vs. t programs used during the various experiments involving parallel oxygen and chlorine evolution.*

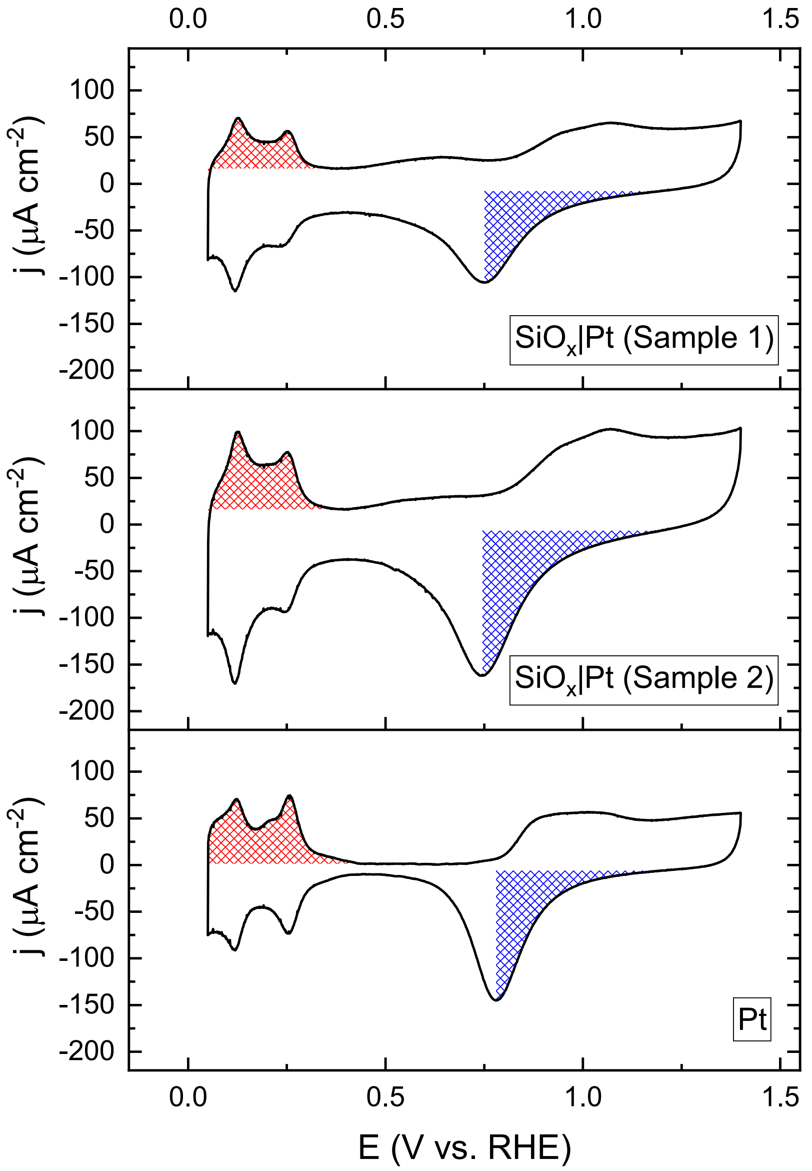


*Figure S 2: Voltammetric characterizations of $SiO_x$|Pt and Pt electrodes, in 0.5 M $KHSO_4$, recorded at 50 mV $s^{-1}$. Charge regions used for comparing hydrogen desorption and $PtO_x$ reduction are illustrated in red and blue, respectively.*

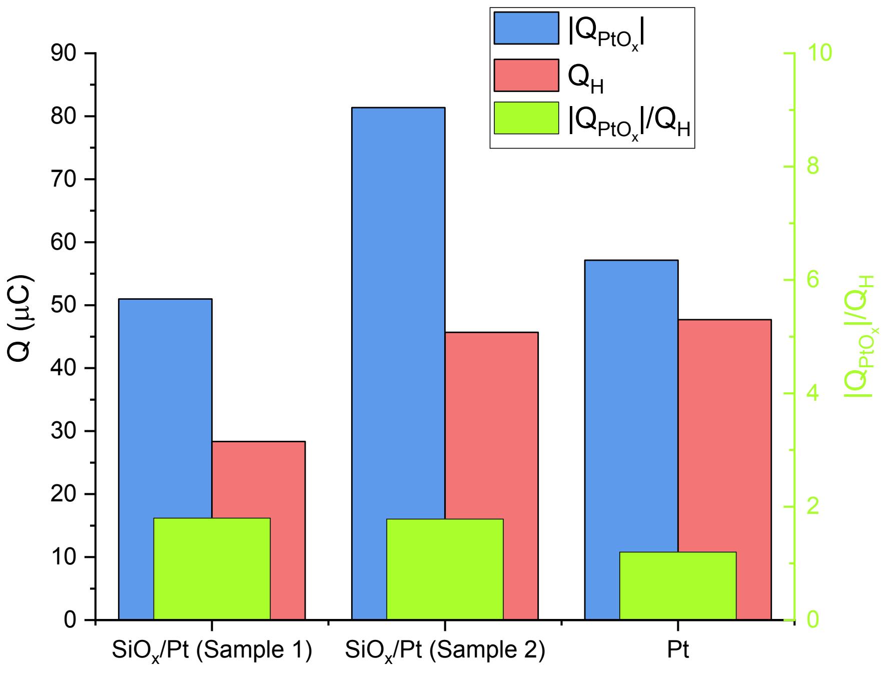


*Figure S 3: Comparison of $Q_H$ and $Q_{PtOx}$, the charges corresponding to hydrogen desorption and $PtO_x$ reduction, respectively. Green bars (values on right axis) show the charge ratios. Data derived from highlighted areas in Figure S 2.*

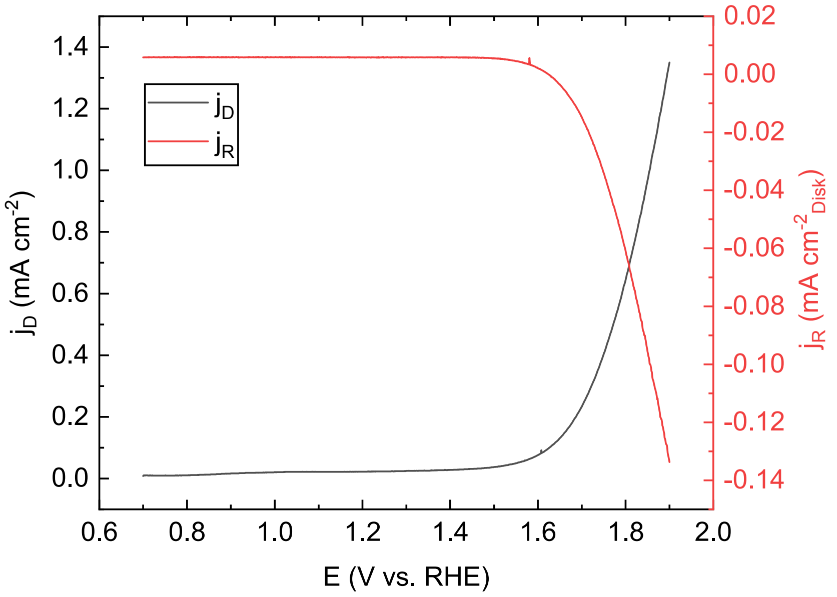


*Figure S 4: Linear RRDE scan on a SiOx|Pt|Ti|GC electrode (Sample 1), recorded at 10 mV $s^{-1}$ in 0.5 M $KHSO_4$. The ring potential was fixed at 0.4 V, so that $O_2$ is detected.*

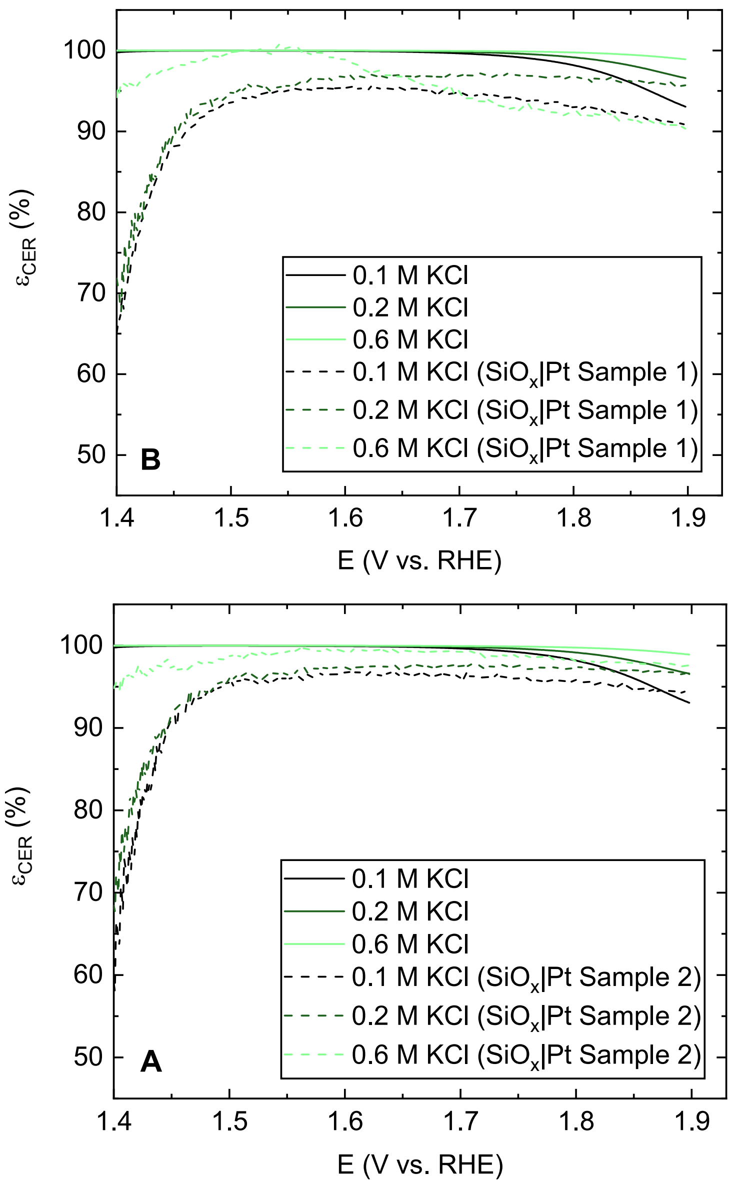


*Figure S 5: Molar CER selectivities versus potential for two SiOx|Pt|Ti|GC electrodes (dotted lines in A and B). Data are shown for three different chloride concentrations, and compared with the bare Pt surface (solid lines). Data for the bare Pt surface were estimated from OER data in absence of chloride.*

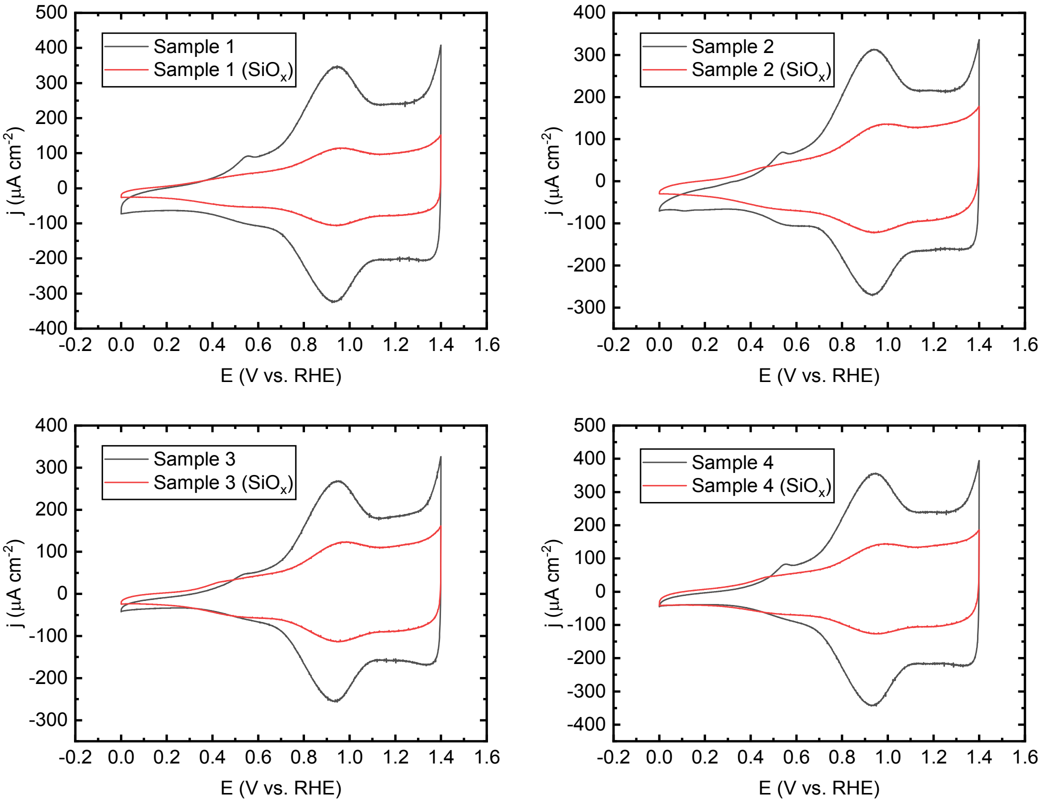


*Figure S 6: Voltammetric characterization of $IrO_x$|GC samples, before and after the $SiO_x$ coating.*

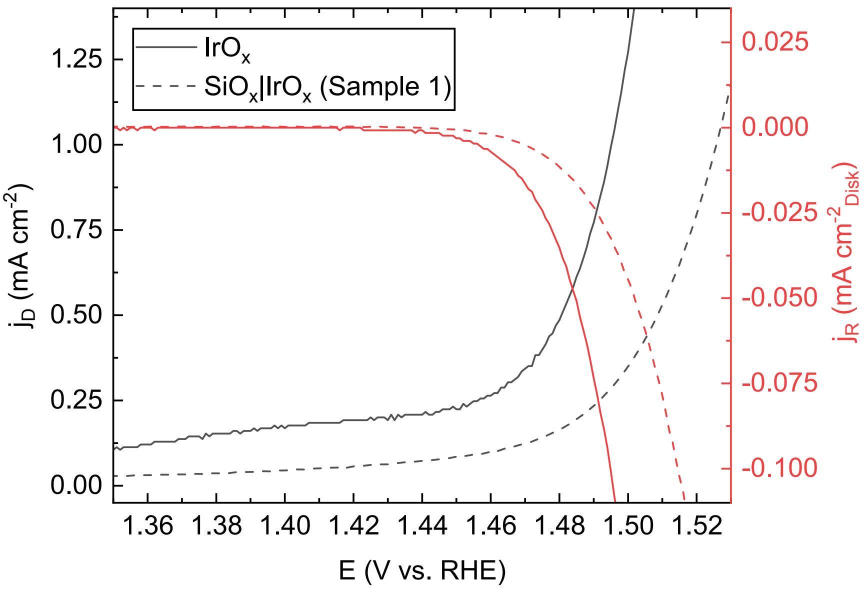


*Figure S 7: Collection experiment of $O_2$ on an $IrO_x$|GC and an $SiO_x$|$IrO_x$|GC samples, as in Figure S 4.*

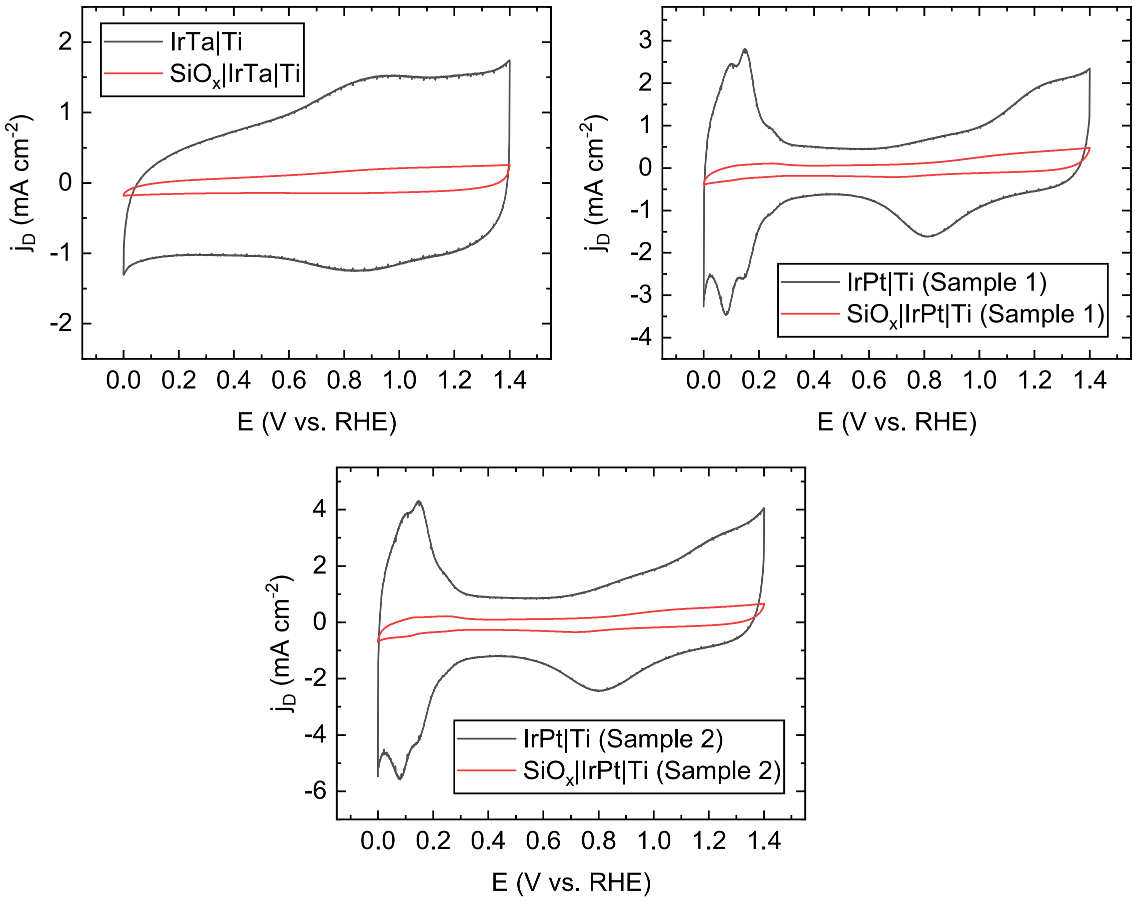


*Figure S 8: Voltammetric characterization of Ti-based anodes, before and after the $SiO_x$ coating. Scan rate: 50 mV $s^{-1}$.*

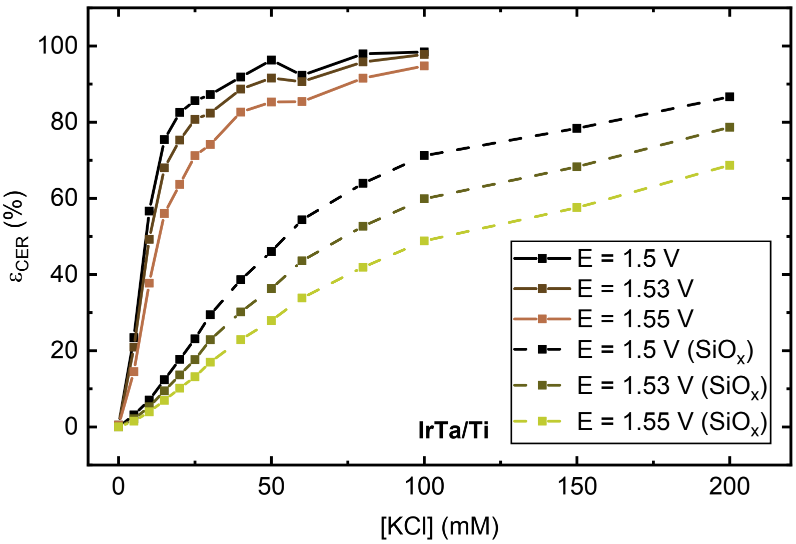


*Figure S 9: Molar selectivity towards the CER as function of chloride concentration for an IrTa/Ti sample, as in Figure 7 in the main text.*

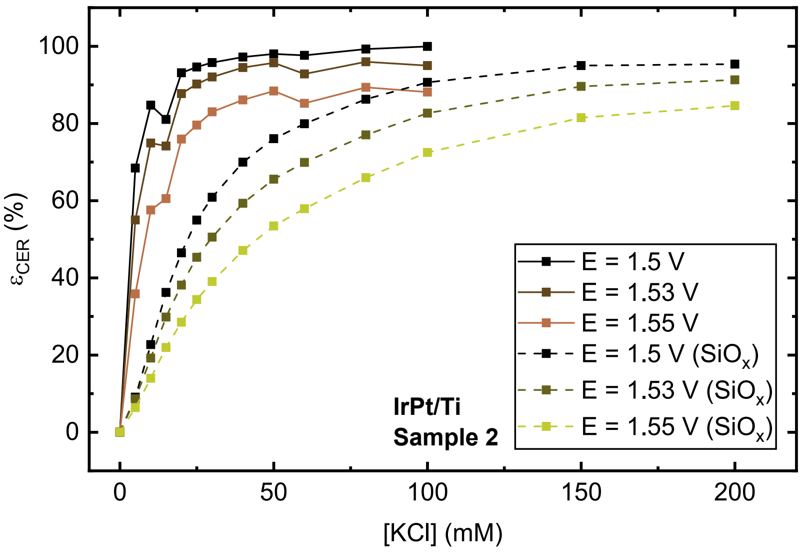


*Figure S 10: Molar selectivity towards the CER as function of chloride concentration for an IrPt|Ti sample, as in Figure 7 in the main text.*

## 2. Kinetic OER and CER data

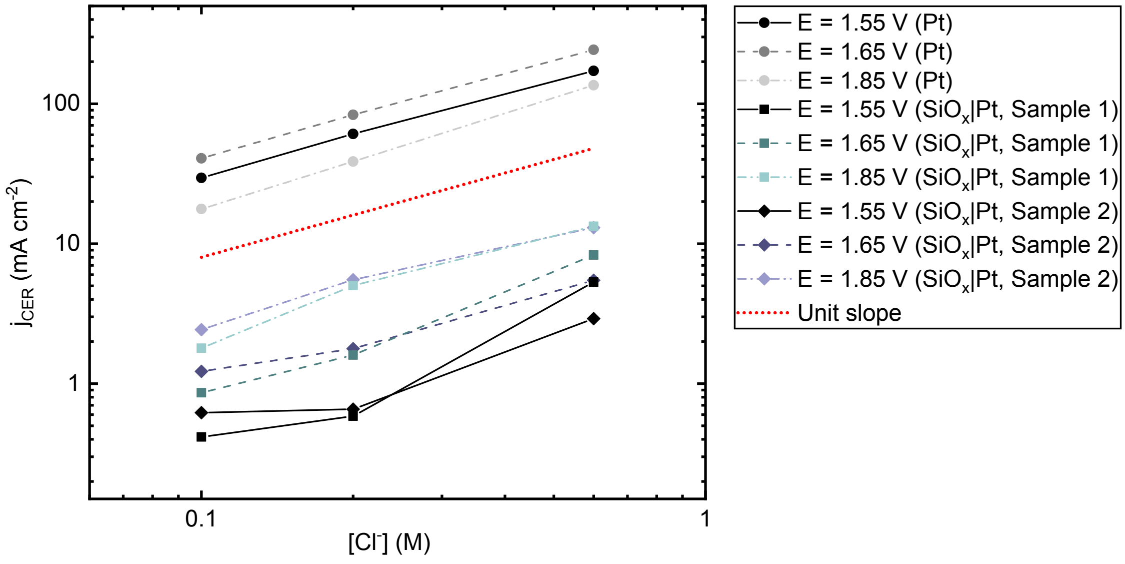


*Figure S 11: Log-log plot of derived CER current densities on various Pt samples, as function of chloride concentration. Data are displayed for three different potentials. Red dotted line shows a reaction order of one (unit slope).*

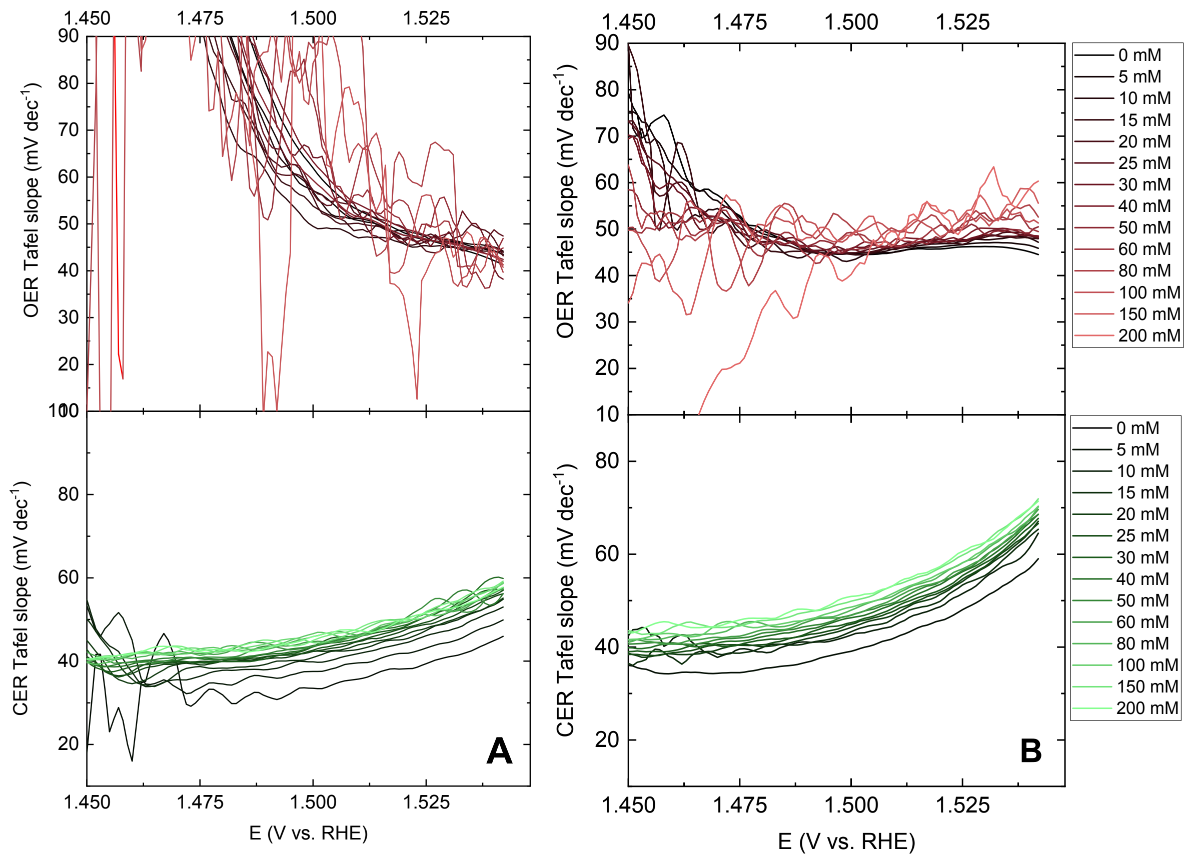


*Figure S 12: Derived Tafel slopes of CER and OER current densities versus potential, on an $IrO_x$|GC sample and an $SiO_x$|$IrO_x$|GC sample (A and B respectively). Values are shown for KCl concentrations ranging between 0 – 200 mM.*

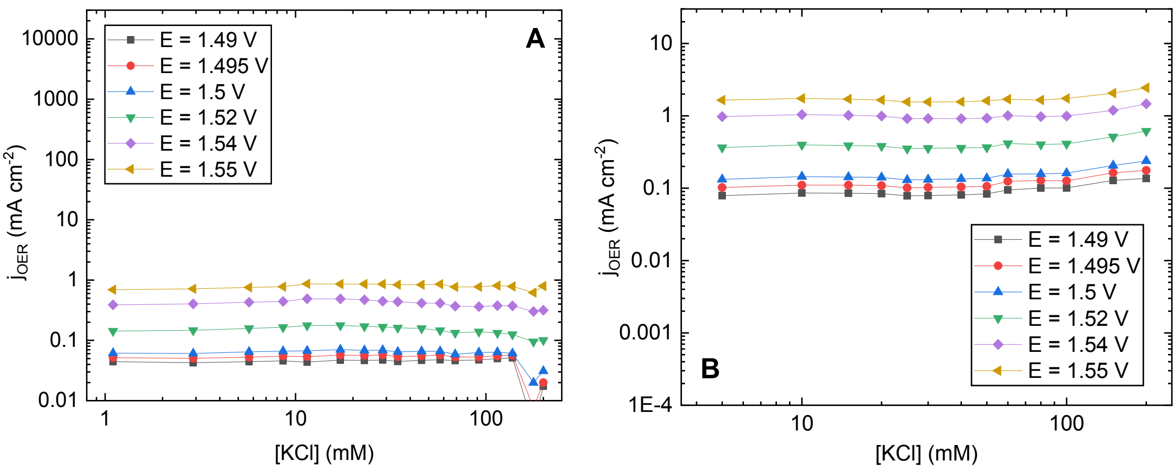


*Figure S 13: Log-log plot of derived OER current densities on an $IrO_x$|GC sample and a $SiO_x$|$IrO_x$|GC sample (A and B respectively). Data are displayed for a series of different potentials.*

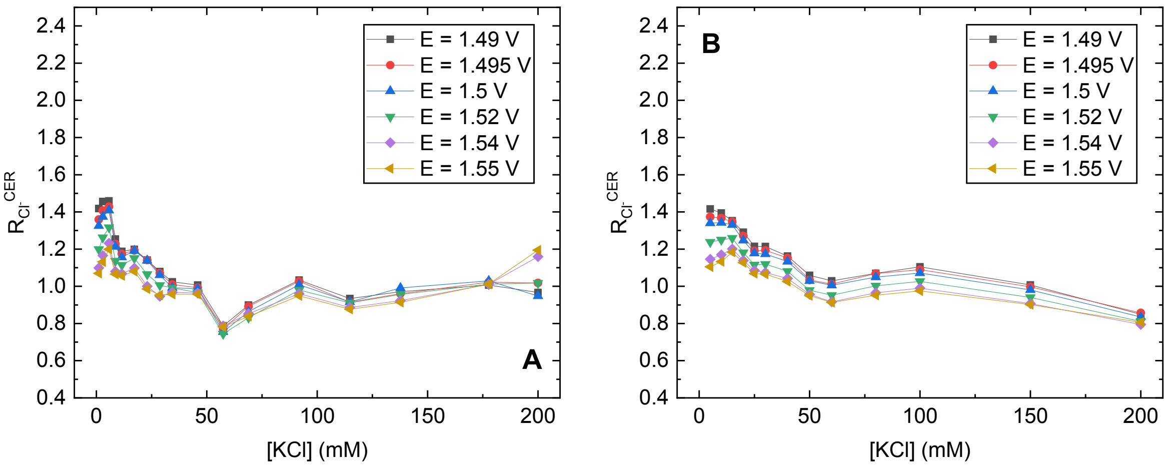


*Figure S 14: CER chloride reaction orders (slopes of log-log plot) versus concentration, derived from an $IrO_x$/GC sample and a $SiO_x$/$IrO_x$/GC sample (A and B respectively). Data are displayed for a series of different potentials.*

## 3. Supplementary SEM/EDS data

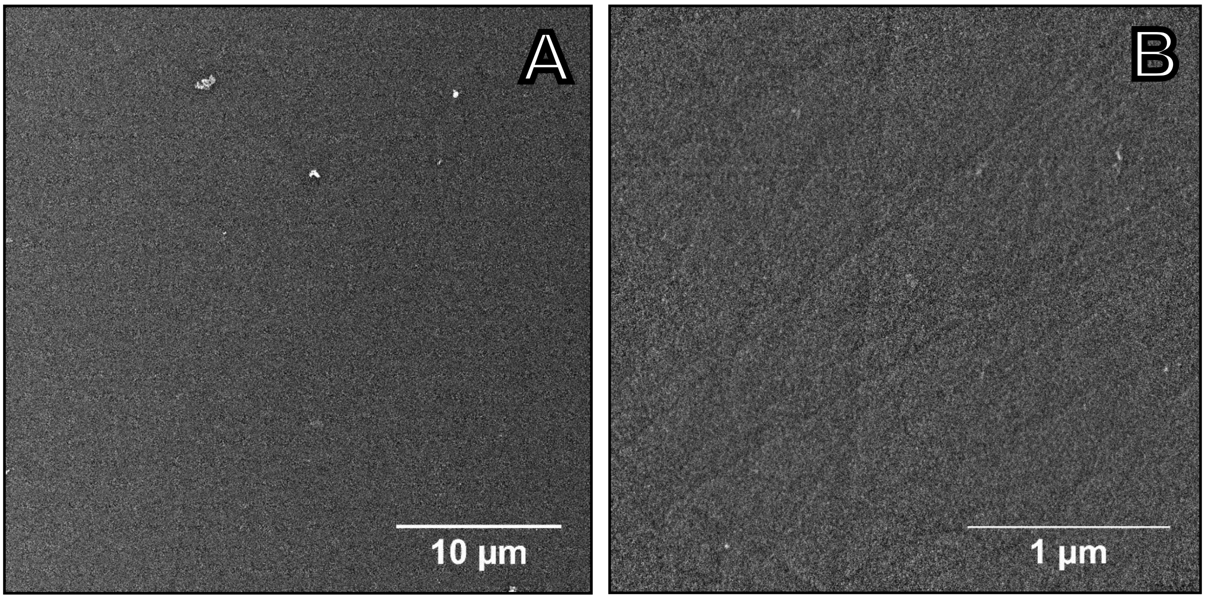


*Figure S 15: SEM micrographs of a $SiO_x$/Pt/Ti/GC electrode surface (including some minor surface impurities in A).*

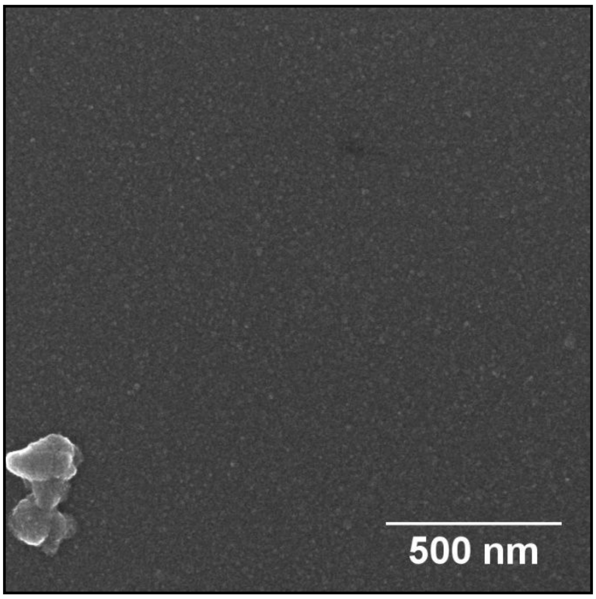


*Figure S 16: SEM micrographs of an $IrO_x$|GC electrode surface, including a typical '$IrO_x$ cluster' (lower-left corner). No $SiO_x$ overlayer is present.*

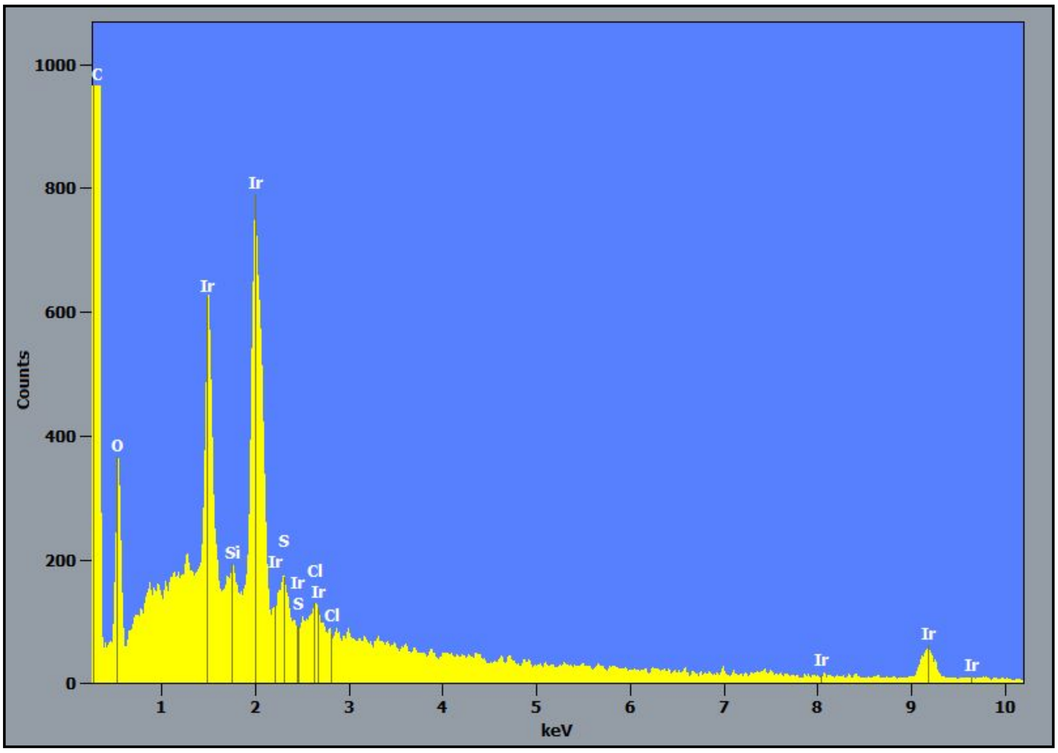


*Figure S 17: EDS spectrum of a $SiO_x$|ir$O_x$|GC electrode surface, showing the peaks of commonly detected elements.*

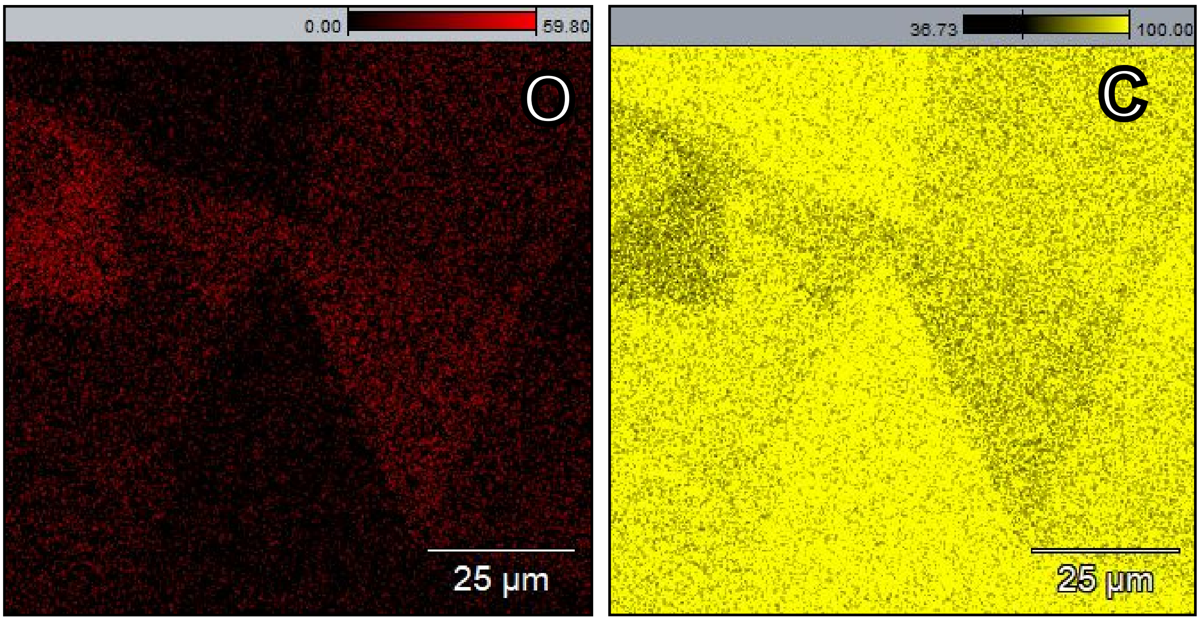


*Figure S 18: Additional EDS elemental maps of O and C, corresponding to the micrograph in Figure 5 in the main text. The C signal is present in large amounts and originates from the bulk GC electrode.*

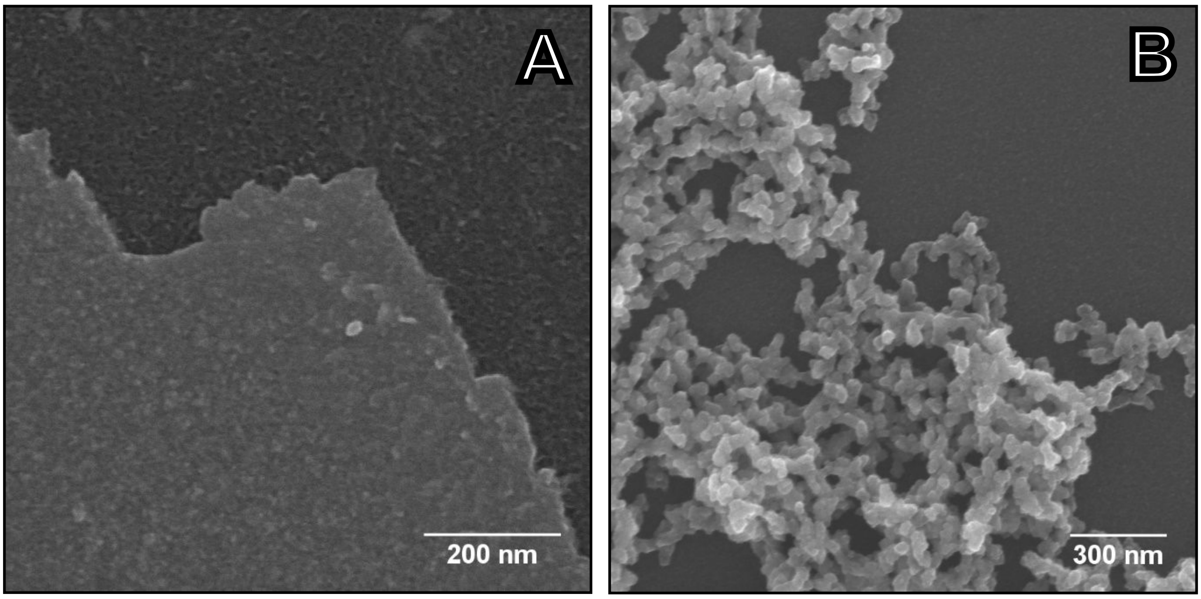


*Figure S 19: Additional SEM electron images of an $IrO_x$|GC surface, after electrochemical experiments (no $SiO_x$ overlayer present). A: Local edge of the 'smooth' $IrO_x$ layer in light grey color, bottom left; the dark-colored bare GC substrate is visible in the top right. B: Close-up of an $IrO_x$ nanoparticle cluster.*

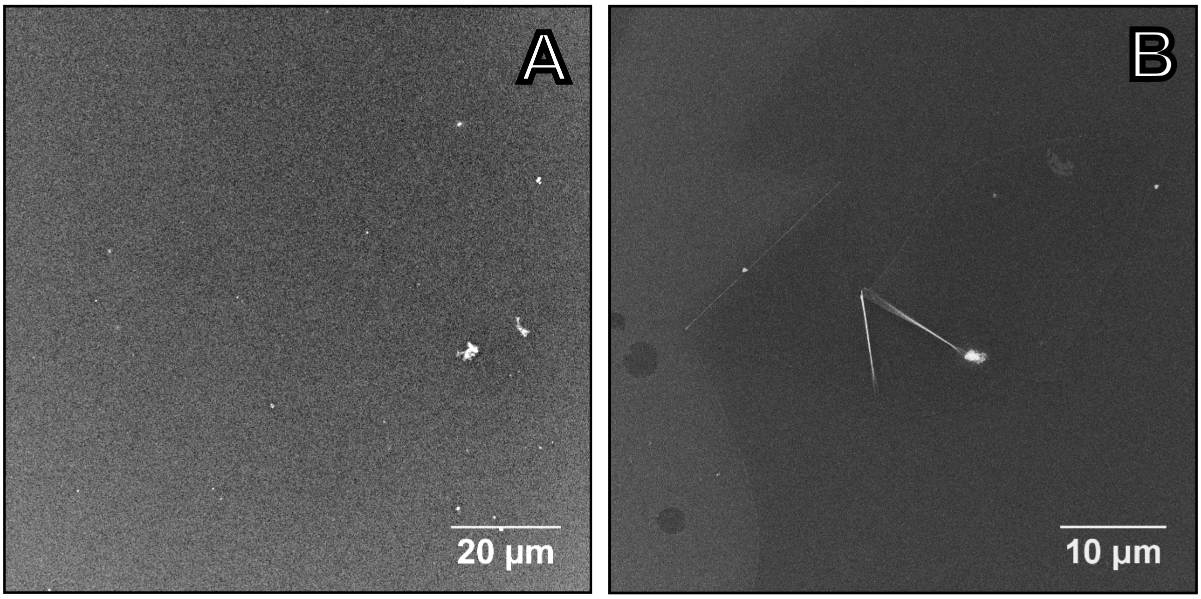


*Figure S 20: Additional SEM electron images of a $SiO_x$|$IrO_x$|GC electrode, after electrochemical experiments. Figure A shows an area where the $SiO_x$ overlayer appears pristine; in Figure B, $SiO_x$ delamination is apparent, similar to Figure 5 in the main text.*

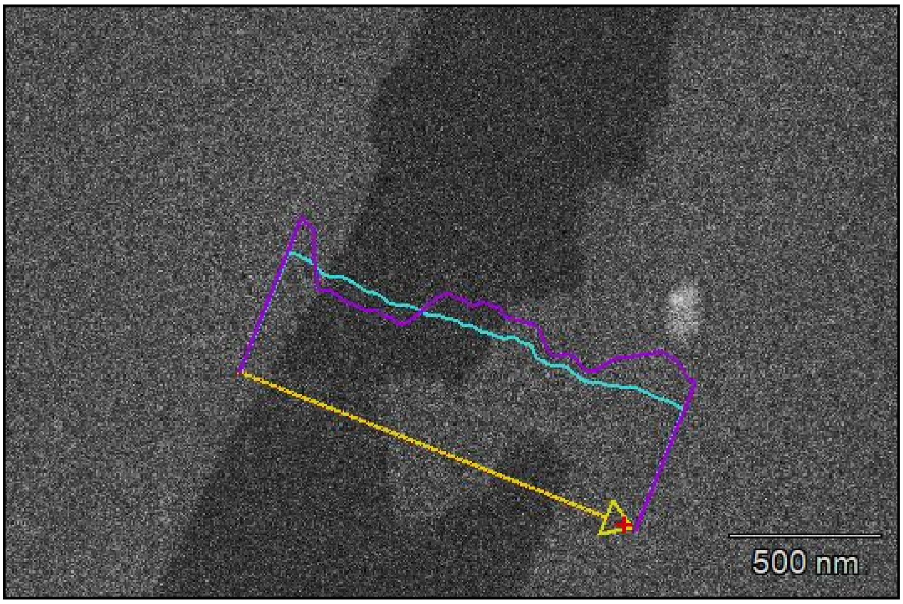


*Figure S 21: SEM|EDS line scan analysis of a $SiO_x$|$IrO_x$|GC surface, after electrochemical experiments. Yellow arrows show scan trajectory with corresponding relative counts of Si (cyan) and Ir (purple). Along the scan, one can observe an inhomogeneous Ir distribution (purple) with an even distribution of Si (cyan) on top.*

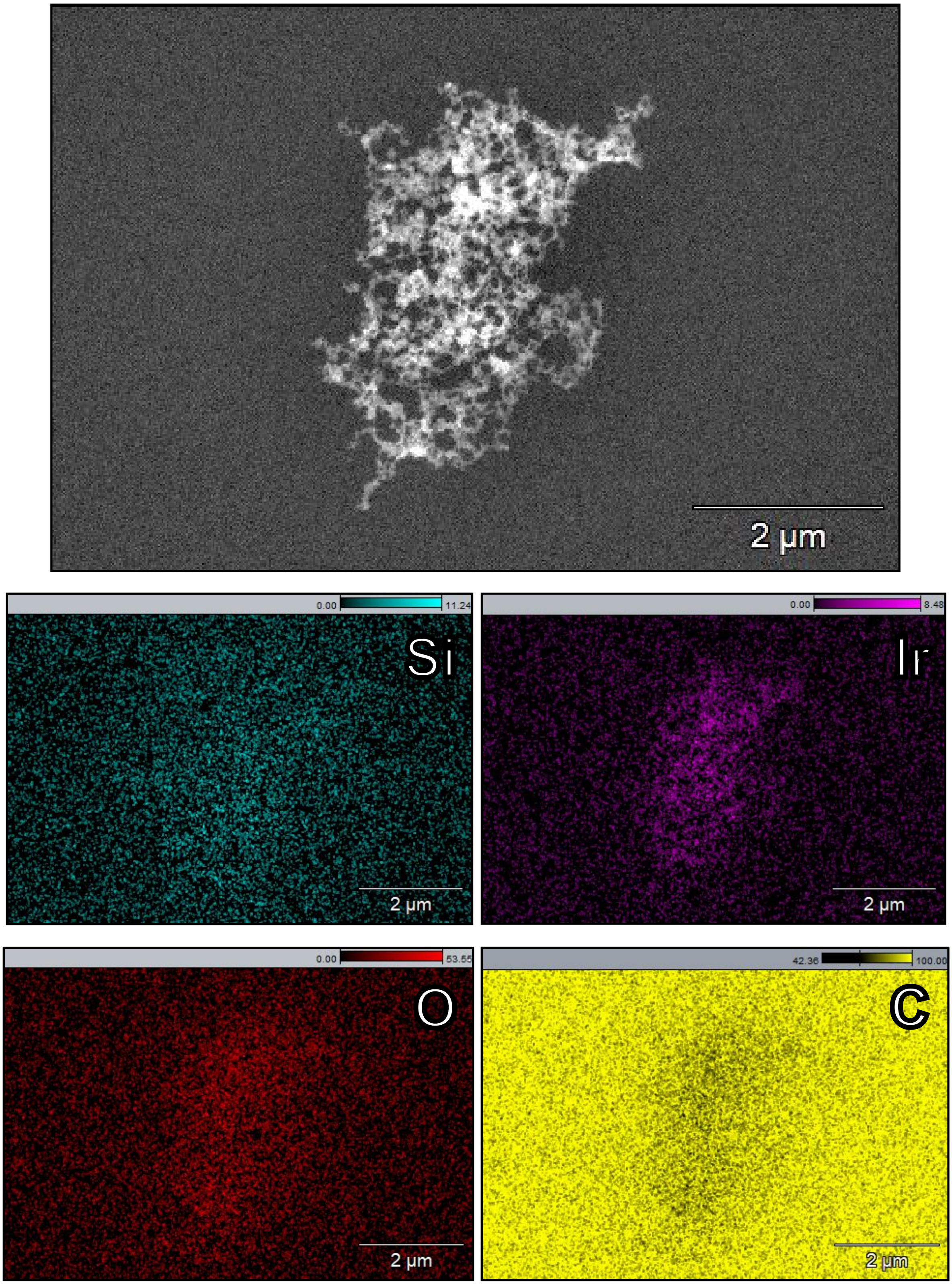


*Figure S 22: Micrograph and EDS elemental mapping of an $IrO_x$ cluster on a $SiO_x$|$IrO_x$|GC electrode, after electrochemical experiments.*

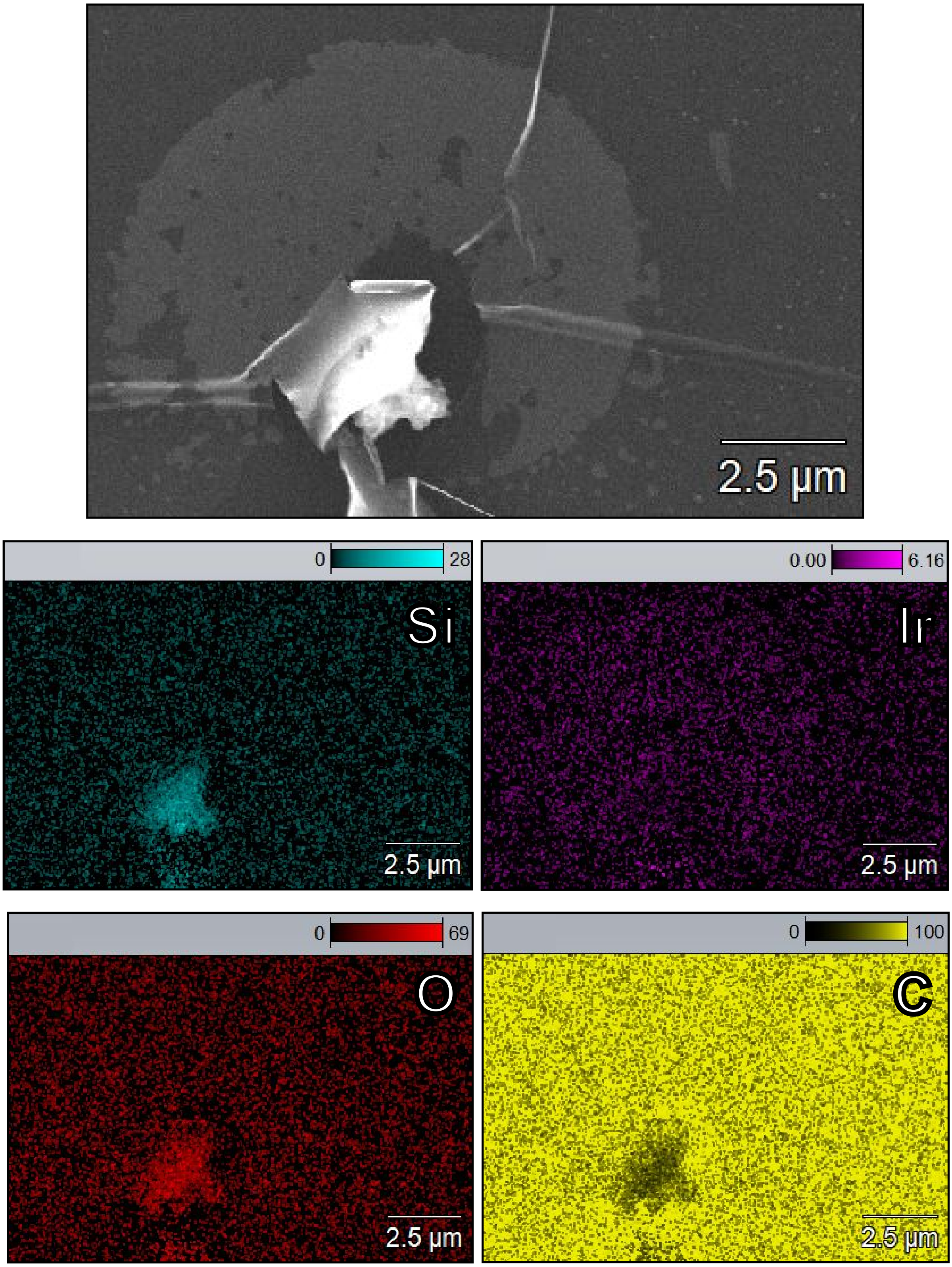


*Figure S 23: Micrograph and EDS elemental mapping of a defect in the $SiO_x$ overlayer on a $SiO_x|IrO_x|GC$ electrode, after electrochemical experiments.*

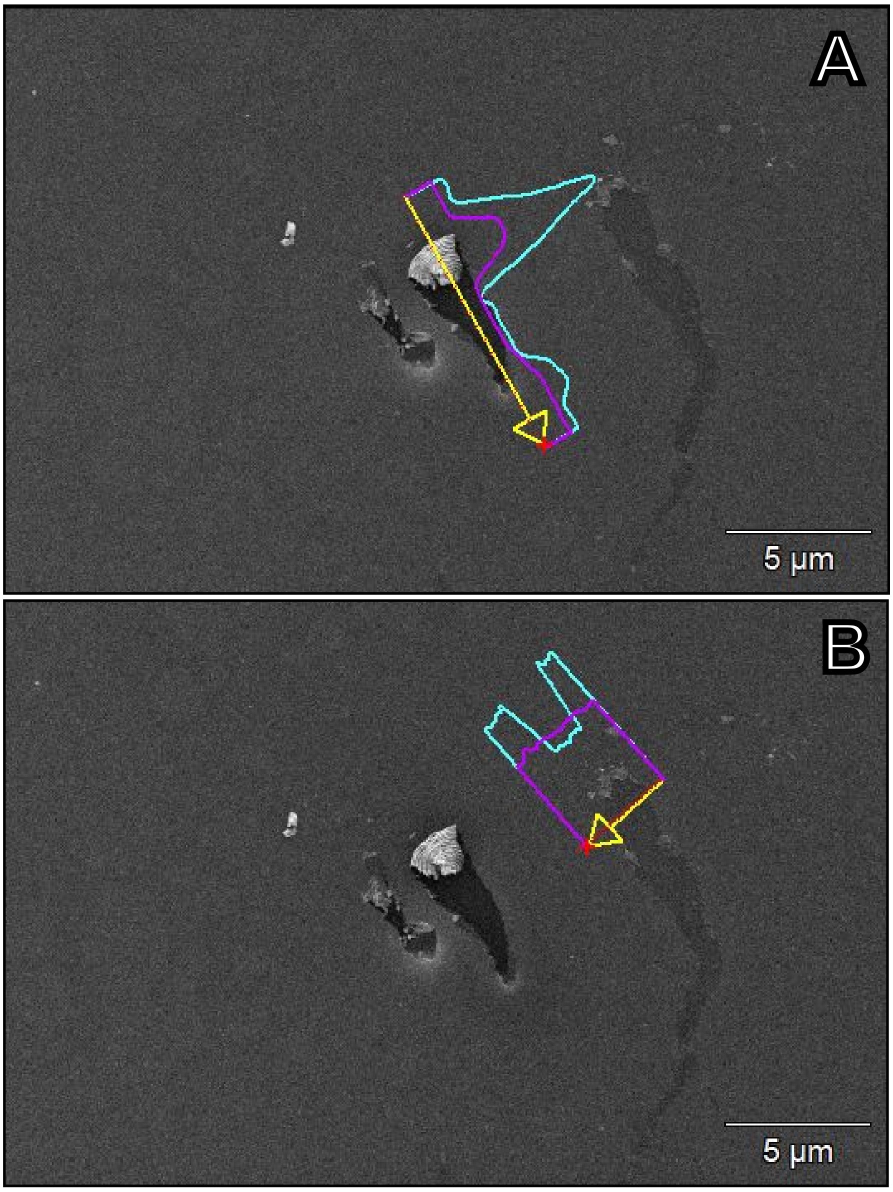


*Figure S 24: SEM|EDS line scan analysis of a $SiO_x|IrO_x|GC$ surface, similar to Figure S 21. Relative amounts of Si and Ir are show in cyan and purple, respectively. In line scan A, a location is investigated where the combined $SiO_x|IrO_x$ film was damaged; material has accumulated around the upper area of the scan trajectory, exposing the bare GC underneath. In B, only the $SiO_x$ overlayer was damaged, as suggested by the even Ir distribution.*

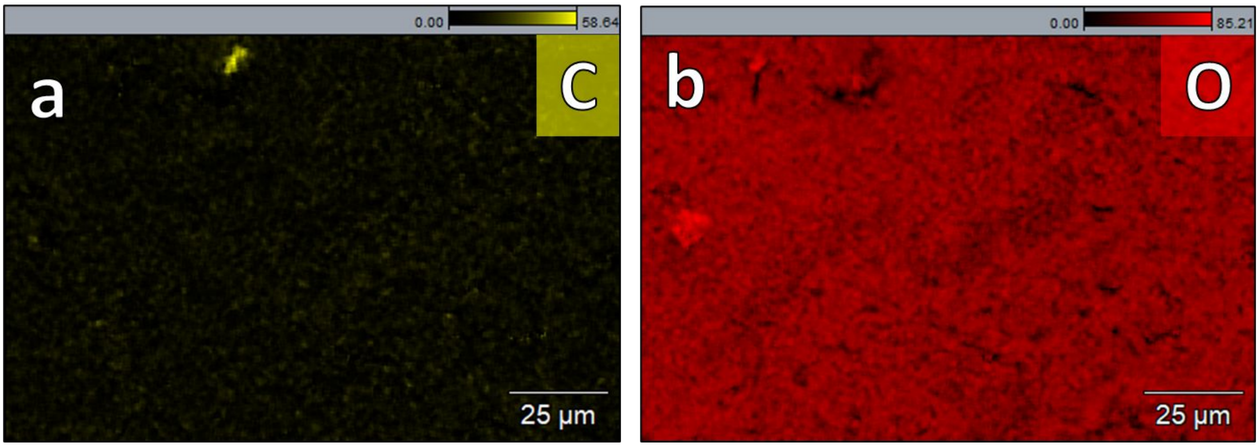


*Figure S 25: Additional EDS elemental maps of C and O of the micrograph in Figure 8 in the main text.*
